# Application of an assay Cascade methodology for a deep preclinical characterization of polymeric nanoparticles as a treatment for gliomas

**DOI:** 10.1080/10717544.2018.1436099

**Published:** 2018-02-07

**Authors:** Cristina Fornaguera, Miguel Ángel Lázaro, Pau Brugada-Vilà, Irene Porcar, Ingrid Morera, Marta Guerra-Rebollo, Cristina Garrido, Núria Rubio, Jerónimo Blanco, Anna Cascante, Salvador Borrós

**Affiliations:** a Sagetis-Biotech, Barcelona, Spain;; b Grup d’Enginyera de Materials (GEMAT), Institut Químic de Sarrià, Universitat Ramon Llull, Barcelona, Spain;; c Institut de Química Avançada de Catalunya (IQAC-CSIC), Barcelona, Spain;; d Centro de Investigación Biomédica en Red en Bioingenierı´a, Biomateriales y Nanomedicina (CIBER-BBN), Barcelona, Spain

**Keywords:** NCL assay cascade protocol, nanoparticle preclinical characterization, research lab results translation, polymeric nanoparticles, paclitaxel, glioblastoma multiforme

## Abstract

Glioblastoma multiforme (GBM) is the most devastating primary brain tumor due to its infiltrating and diffuse growth characteristics, a situation compounded by the lack of effective treatments. Currently, many efforts are being devoted to find novel formulations to treat this disease, specifically in the nanomedicine field. However, due to the lack of comprehensive characterization that leads to insufficient data on reproducibility, only a reduced number of nanomedicines have reached clinical phases. In this context, the aim of the present study was to use a cascade of assays that evaluate from physical-chemical and structural properties to biological characteristics, both *in vitro* and *in vivo*, and also to check the performance of nanoparticles for glioma therapy. An amphiphilic block copolymer, composed of polyester and poly(ethylene glycol; PEG) blocks, has been synthesized. Using a mixture of this copolymer and a polymer containing an active targeting moiety to the Blood Brain Barrier (BBB; Seq12 peptide), biocompatible and biodegradable polymeric nanoparticles have been prepared and extensively characterized. *In vitro* studies demonstrated that nanoparticles are safe for normal cells but cytotoxic for cancer cells. *In vivo* studies in mice demonstrated the ability of the Seq12 peptide to cross the BBB. Finally, *in vivo* efficacy studies using a human tumor model in SCID mice resulted in a significant 50% life-span increase, as compared with non-treated animals. Altogether, this assay cascade provided extensive pre-clinical characterization of our polymeric nanoparticles, now ready for clinical evaluation.

## 1. Introduction

The term nanomedicine defines any nanoscale tool, typically with at least one dimension in the submicrometer range (<1000 nm), that is intended for the diagnosis, prevention, or treatment of diseases (Duncan & Gaspar, 2011; Gaudin et al., 2015). This term appeared in the nineties and since then, nanomedicine research studies have experienced an exponential increase (Pinto Reis et al., 2006; Dobrovolskaia & McNeil, 2007; Bazile, 2014). The pharmaceutical industry’s early acknowledgement of the high potential of nanomedicines resulted in the development of multiple studies aiming at reaching the market in a near future. However, their translation to clinical assays has been markedly limited (Venditto & Szoka, 2013; Ge et al., 2014; Venkatraman, 2014; Mitragotri et al., 2015). Poor clinical performance could be attributed, among many other factors such as expensive production and lack of effectiveness, to lack of effective characterization and bio-safety guarantees.

In order to circumvent some of these gaps between laboratory research and pharmaceutical application, the Nanotechnology Characterization Laboratory (NCL) implemented an assay-cascade protocol that consists on a series of recommended tests for extensive characterization of nanomaterials prior to clinical studies (Hall et al., 2007; Dobrovolskaia & McNeil, 2013). This approach evaluates physicochemical and pharmacodynamics parameters as well as safety and performance characteristics of the nanomedicine ensuring product reproducibility and fulfillment of quality, safety, and efficacy; regulatory requirements to reach the clinic market. Although this assay-cascade protocol is not a requirement for the approval of regulatory entities (i.e. Food and Drug Administration (FDA) and European Medicines Agency (EMA)), the authors considered it an appropriate guideline to follow when designing novel nanomedicines to achieve the approval of regulatory agencies.

In our lab, polymeric nanoparticles have been previously synthesized for many purposes. In the present work, polymeric nanoparticles have been designed for the treatment of glioblastoma multiforme (GBM), the most malignant brain tumor ( McGirt et al., 2009a,b; Zhang, 2011; Zhang et al., 2011; Chaichana et al., 2013,2014; Ostrom et al., 2014). Existent therapies against GBM comprise tumor resection followed by radiotherapy and/or systemic and local administration of chemotherapy. However, chemotherapeutics have severe side effects and low effectiveness, all together resulting in poor median survivals of only 12–15 months. Paclitaxel (PTX) is one of the most effective cancer chemotherapeutic agents known, highly efficacious in the treatment of different cancers (Crown et al., 2004; McGrogan et al., 2008). However, it presents many problems regarding its *in vivo* use. First of all, PTX solubility in aqueous solvents is very low, thus limiting the possible dose to be administered. Therefore, previous studies have used the Cremophor EL surfactant to enhance its solubility. However, this surfactant presents dose-limiting toxicity problems that have already not been circumvented (Miele et al., 2009). Second, in brain tumor patients, it has shown only moderate activity (Chamberlain & Kormanik, 1995), mainly due to low Blood Brain Barrier (BBB) permeability (Kabanov & Gendelman, 2007; Zlokovic, 2008; Pardridge, 2012; Kreuter, 2014). Although there are many studies demonstrating the impairment of the BBB in the presence of gliomas, PTX is not able to produce a therapeutic effect to the brain, mainly due to its excretion by the P-glycoprotein (P-gp) pump; whose activity has been demonstrated even in leak BBBs (Régina et al., 2009). The firsts treatments with PTX for gliomas were very invasive, since they consisted on the intratumor administration of the drug, showing a high anti-tumor response, but were also associated with severe side effects (Régina et al., 2009). Thus, there is an urgent need to develop new treatments against GBM. Research labs experience in the last decades confirms polymeric nanoparticles as appropriate drug delivery systems (DDS) to diagnose and treat cancer, specifically, brain cancer, due to the multiple advantages they represent; e.g. the possibility to functionalize them with peptides targeting specific receptors of the BBB, such as the Seq12 Sagetis proprietary peptide (Borrós et al., 2014), for the BBB crossing and the active targeting to the brain without being substrate of the P-gp pump. Nevertheless, as stated above, their translation to clinical trials is markedly limited (Gref et al., 1995; Béduneau et al., 2008; Mura & Couvreur, 2012; Neha et al., 2013; Alyautdin et al., 2014; Kreuter, 2014).

In the current work, we demonstrate that the application of a NCL-like assay cascade protocol enables the characterization of polymeric nanoparticles, enabling their transference to clinical experimentation; thus ensuring their quality, safety, and efficacy.

## 2. Experimental part

### 2.1. Materials

1.8-Octanediol (98%) was purchased from Sigma-Aldrich (Germany) and poly(ethylene glycol) (α-thio ω-carboxy PEG) (Mw 3000 Da) was purchased from IRIS biotech(Germany). Glutaryl dichloride (99%) was obtained from Sigma-Aldrich and was distilled under reduced pressure prior to use. PTX (≥97%) was provided by Yunnan Hande Bio-Tech CO, LTD (P.R. China). Seq12 peptide (patent WO2013IB60137 20131114 (Borrós et al., 2014) was obtained from GL Biochem (P.R. China). FBS was purchased from Lonza (Suisse), L-glutamine and Penicillin/Streptomycin were provided by Labclinics (Spain). All other chemicals of analytical grade were purchased from Sigma (Sigma-Aldrich, Germany).

### 2.2. Methods

#### 2.2.1. Synthesis of P and 2P block copolymers

**Figure 1.**
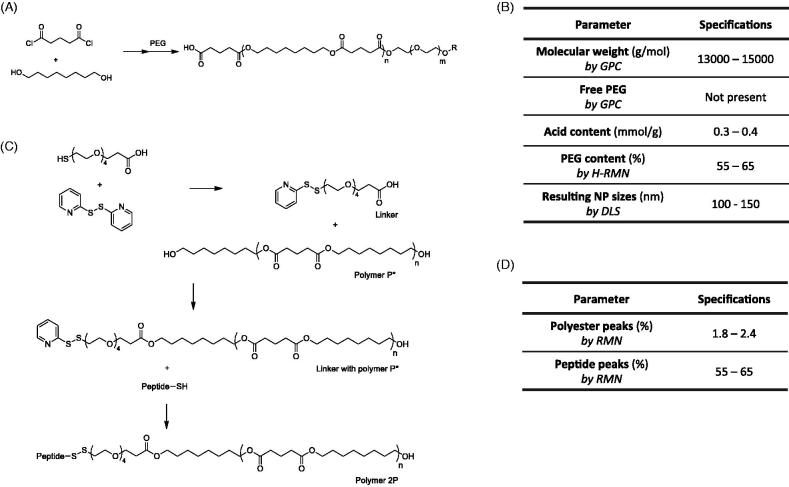
(A) Schematic representation of P polymer; (B) Quality specification criteria that a synthesized polymer P must accomplish to consider it appropriate for the desired use; .(C) Schematic representation of 2 P polymer synthesis; and –(D) Additional quality specification criteria required for adequacy of 2 P polymer.

The P polymer synthesis (Figure 1(A)) started with the addition of 30 g of 1,8-octanediol (0.21 mol) in a thermojacket reactor, with an exit to a 1 M sodium hydroxide solution; and it was cooled down to 10 °C. 32 mL of glutaryl dichloride (0.25 mol) were added and the mixture was stirred under argon flow at 70 °C. After 30 min, 74 g of PEG (0.035 mol) were added and the mixture was stirred for another 30 min. The reaction mixture was then cooled to 31 °C and 20 mL of acetone were added and mixed for five additional minutes. Next, 300 mL of water were added and the mixture was stirred for 30 min. The resulting suspension was centrifuged at 6000 rpm for 20 min and the supernatant was discarded. This step was repeated once and the polymer was washed with 300 mL water. Finally, the pellet was freeze-dried and the polymer was stored at −20 °C.

The polymer 2P is a modification of the polymer P; having the same backbone structure and a terminal peptide, the Seq12 (Figure 1(B)). Briefly, 2.58 g of 1,8-octanediol (18 mmol) were mixed with 2.0 g of glutaryl dichloride (12 mmol). After 1 h stirring at 70 °C, the mixture was cooled to room temperature and 15 mL of diethyl ether was added. This product was washed three times with methanol. The organic phase (diethyl ether) was dried and then freeze-dried. This product was called polymer P*.

The linker necessary to attach the peptide was obtained in parallel, by mixing 0.53 ml of acetic acid to a solution of 70 mg of 2,2′-dithiopyridine (0.32 mmol) in 4 mL dry tetrahydrofurane under inert atmosphere (argon). Following, a solution of 500 mg α-thio ω-carboxy PEG (0.16 mmol; Mw = 3300 Da) were stepwise added in dry 1.3 mL tetrahydrofurane under inert atmosphere (argon). The reaction mixture was stirred for two days at room temperature and was later added to 10 ml of diethyl ether. Then, the suspension was centrifuged at 4000 rpm for 10 min and the solvent was removed. Five milliliterS of diethyl ether was added to the solid and after a short vortex agitation, the solution was centrifuged at 4000 rpm for 10 min. Finally, the solvent was removed by evaporation under reduced pressure and the obtained product was dried overnight.

Thirteen milligramS of dicyclohexylcarbodiimide (0.064 mmol) and 5 mg of N,N′-dimethylaminopyridine (0.038 mmol) were added to a solution of 100 mg linker (0.032 mmol) in 0.8 mL dry dichloromethane. The reaction mixture was stirred for 1 h at room temperature. Two hundred milligram of Polymer P* in 0.5 mL dry dichloromethane was added to the reaction and stirred at room temperature for 20 h. The solvent was reduced under vacuum and 10 mL diethyl ether were added to the residue. The suspension was centrifuged at 4000 rpm for 10 min and the solvent was removed. Five milliliterS of diethyl ether was added to the solid and after a short vortex agitation, it was centrifuged at 4000 rpm for 10 min. The solvent was removed and the obtained product was dried overnight. Eighty-eight milligramS of Seq12 peptide were added to 242 mg of the above product in 2.1 mL dry dimethylformamide. The reaction was stirred for three days at room temperature and was later added to 10 mL diethyl ether. The suspension was centrifuged at 4000 rpm for 10 min and the solvent was removed. Finally, 5 mL diethyl ether were added to the solid and after a short vortex agitation was centrifuged at 4000 rpm for 10 min. the final product was obtained and was dried overnight.

Both polymers were characterized by gel permeation chromatography (GPC), proton nuclear magnetic resonance (^1 ^H-NMR), and acid titration and peptide quantification (see detailed techniques in explanation in S1 of the Supplementary material).

#### 2.2.2. Preparation of polymeric nanoparticles

Nanoparticles were synthesized using the nanoprecipitation method (Fessi et al., 1989; Vauthier & Bouchemal, 2009; Neha et al., 2013). Two sizes of nanoparticle batches were synthesized: small (20 mg of solid content) and large batches (800 mg of solid content).

For the preparation of a small batch, 0.6 mg polymer 2 P were dissolved in 50 μL dimethyl sulfoxide (DMSO), vortexed and sonicated until complete dissolution. 19.4 mg polymer P were dissolved in 550 μL acetone and vortexed. Polymer 2 P was transferred to polymer P solution and mixed. Four hundred microliter of PTX solution 1 mg/mL in acetone were also added. This solution was added stepwise (50 μL/min flow rate) to 800 mL milliQ ultrapure water(Millipore, Billerica, MA) to perform nanoprecipitation. Following, the dispersion was centrifuged (6000 rpm, 20 min) using Amicon Ultra-15 Centrifugal Filter Units with a 100 kDa cutoff regenerated cellulose membrane (Millipore) and the pellet was redispersed in 1 mL milliQ water.

For the preparation of a large batch, 24 mg of polymer 2 P were dissolved in 1.5 mL DMSO, vortexed and sonicated for 15 min. 776 mg of polymer P and 16 mg PTX were dissolved in 38.5 mL acetone and stirred until complete dissolution. Polymer 2 P was transferred to polymer P solution and mixed. This solution was added stepwise (50 μL/min flow rate) to 800 mL milliQ water to perform nanoprecipitation. Following, nanoparticle dispersion was filtered using tangential flow filtration (TFF; 300 kDa MWCO regenerated cellulose membranes) to filter and concentrate nanoparticles to around 10 mL. Finally, TFF was used to transfer nanoparticles to a solution of 10 wt% sucrose to ensure physiological osmolality.

Synthesized nanoparticles were characterized by means of dynamic light scattering (hydrodynamic size, polydispersity, and surface charge – protocols PCC1 and PCC2 from the NCL assay cascade) and electron microscopy to determine size, morphology, and surface charge; osmolality was also determined and ultra-high performance liquid chromatography (UPLC) was used to determine drug content and peptide quantification (as detailed in explanation S2, in the Supplementary material).

#### *2.2.3.* In vitro *safety and toxicity evaluation*

##### 2.2.3.1 Endotoxin determination

To discard the presence of endotoxin contamination, the end-point chromogenic LAL assay (modification of the STE-1.1 protocol from the NCL assay cascade) has been used, following the indications of the supplier (Lonza).

##### 2.2.3.2 Cytotoxicity studies

Cell viability was assessed with the 3-(4,5-dimethylthiazol-2-yl)-2,5-diphenyltetrazolium bromide (MTT) colorimetric assay (protocol modified from the GTA-1/2 protocols of the NCL assay cascade). Three cell lines were used: U87-MG, a glioblastoma; BBMVECs bovine brain microvascular endothelial cells (BBMVECs), and rat cortical astrocytes(RCA) models of normal neural cells. For each assay, 3000 cells/well were seeded on a 96-well plate in 100 μL of DMEM and cultured for 24 hours at 37 °C under 5% CO_2_ atmosphere. Then, samples of nanoparticles and controls were added at the required concentrations. Cells were incubated during the indicated times. The MTT reagent was added at a final concentration of 0.5 mg/mL in phosphate-buffered saline (PBS) and were incubated for 3 h at 37 °C; thereafter, the medium was withdrawn and 200 μL of DMSO were added to dissolve the formazan crystals. The plate was stirred for 15 min at room temperature. Absorbance was measured at *λ* = 570 nm with the SpectraMax M5 spectrophotometer (Molecular Devices, Sunnyvale, CA).

##### 2.2.3.3 Hemolysis determination

Erythrocytes (RBC) were obtained by centrifugation from mouse whole blood (10 min, 867 g). Supernatants were discarded and the erythrocyte pellet were resuspended in PBS to remove traces of plasma. The erythrocyte suspension was dispersed in PBS, at a concentration of 8 x 10^9^ cells/mL. The hemolytic activity was assessed using a spectroscopic procedure, following the protocol from the NCL assay cascade with slight modifications (Dobrovolskaia et al., 2008; McNeil, 2011; Dobrovolskaia & McNeil, 2013). The percent of hemolysis was spectroscopically assessed by comparing the absorbance (*λ* = 540 nm) of the samples with positive (10 wt% Triton X100) and negative (PBS) controls. The results are expressed as the percent of hemolysis caused.

##### 2.2.3.4 Seq12 peptide BBB penetration assay

Seq12 BBB penetration capacity was assayed using a co-culture model of BBB comprising of low passaged BBMVECs grown on a collagen coated Transwell insert membrane, and RCAs (astrocytes) seeded at the bottom of the well (see Supplementary Figure S1). The properties of the BBB model were characterized to confirm its suitability (see Explanation S3 from Supplementary material).

The BBB penetrating angiopep-2 peptide (Régina et al., 2009) was used as a positive control reference for transport experiments; performed in order to determine the BBB crossing levels of Seq12 peptide. Also, a scrambled peptide (SCRMBL) sequence was also tested as a negative control. Briefly, equal molecular amounts of each peptide were added into the luminal compartment of each insert and incubated for one hour at 37 °C and 5% of CO_2_. Samples were collected from both compartments, at the beginning and at the end of the experiment. Fluorescence levels of 5-Carboxytetramethylrhodamine (5-TAMRA) dye was used to label peptides and these peptides were measured using a multi-well plate reader. Mass balance of each peptide was calculated using Equation (1). Peptide amounts can be extrapolated from a calibration curve using known amounts of fluorescently-labeled peptides. Finally, the results were expressed as comparisons with the passage across an insert without cells (taking this value as the 100% of crossing).

(1) $$MB\left(\%\right)=\frac{\begin{array}{l} Peptide\;amount\;\left(Luminal,\;t60\right)\;\\ +\;Peptide\;amount\;\left(Abluminal,\;t60\right) \end{array}}{Peptide\;amount\;(lum,\;t0)}\;$$

A competition assay was performed to demonstrate that the Seq12 peptide interacted with the low density lipoprotein receptor-related protein 1 (LRP-1) receptor. In particular, fluorescently labeled testing peptides (Seq12 and angiopep-2 as positive control) were mixed with a fixed concentration of unlabeled angiopep-2, which has been extensively reported to bind the LRP-1 receptor (Demeule et al., 2008; Régina et al., 2009). Results are presented as decrease of the percentage of BBB crossing when angiopep-2 is present.

#### *2.2.4.* In situ *brain perfusion study*

Two groups of male Wistar rats of around 500 g were randomized and administered: free PTX (dissolved in a solution of 50/50 v/v Cremophor EL/Ethanol) or PTX-loaded NPs. Each animal received 1 mg PTX (5 mL/animal). Brain perfusion experiments were performed following the protocol of Boje (2002). In brief, the perfusion solution was connected with the cardiac ventricles and the solution was infused at 5 mL/min. Later, the brain was removed and the capillaries were depleted. The tissue was homogenized with lysis buffer and centrifuged at 5400 g for 15 min at 4 °C. The concentration of PTX in the supernatant was analyzed by UPLC, as described in the Supplementary material (Explanation S2). Results are given in brain uptake (mL/g) and *K_in_* (mL/g·s). While brain uptake is a static measure of the volume of NPs that have entered the brain per mass of brain tissue, *K_in_* refers to the kinetics of the organ clearance; the unidirectional influx constant from plasma to brain (Bickel, 2005). Statistical analysis was performed using Prism (GraphPad software, La Jolla CA). Differences between experimental groups were analyzed by one -way analysis of variance (ANOVA) with *post-hoc* Tukey’s honest significant difference (HSD) test.

#### *2.2.5.* In vivo *preclinical evaluation*

Adult 6–8 weeks old SCID mice were kept under pathogen-free conditions in laminar flow boxes. Animal maintenance and experiments were performed in accordance with established guidelines of Catalan Government and protocol number 4565 approved by Direcció General del Medi Natural, Generalitat de Catalunya. Final point criteria for animal sacrifice are specified in this protocol (see Explanation S4, Supplementary material).

Animal experiments were performed as described previously (Alieva et al., 2012; Bagó et al., 2013). Briefly, animals were anesthetized by intraperitonea**l** (i.p.) injections of 3.3 mg/kg xylazine (Rompun, Bayer DVM) and 1.39 mg/kg ketamine (Imalgene 100, Merial Laboratorios)). Subsequently mice were mounted in a stereotactic frame (Stoelting, Wood Dale, IL)and 1 x 10^5^ U87-MG cells previously transduced with luciferase-containing lentivirus (Pluc-G-U87-MG cells) were stereotactically implanted. Cell suspension was injected at a 0.6 μL/min rate using a Hamilton syringe series 700 (detailed procedure on Explanation S5, from Supplementary material). One week post implantation, bioluminiscence images (BLI) were acquired to monitor tumor development and animals were randomly divided into two groups: NP and PTX-loaded NPs (*n* = 10 each group). On the following day, NP treatment was initiated (*t* = 0), by intravenous injection of 600 mg/kg NPs (10.8 mg/kg PTX dose). Injections were repeated every three days during a 21 days period. Weekly BLI was performed to follow tumor growth during the experiment. For imaging, mice were anesthetized and then injected i.p. with 150 µl of luciferin (Regis Technologies, Morton Grove, IL;16.7 mg/ml in saline). Animals were placed in the detection chamber of the high efficiency ORCA-2BT Imaging System (Japan) and images were acquired from the dorsal direction. Quantification and analysis of photons recorded in images was done using the Wasabi image analysis software (Hamamatsu Photonics, Japan). Briefly, light events were calculated and expressed as photon events (PHC) after discounting the background noise. The net number of PHCs in the area of interest was calculated using the formula: PHCs = (total number of PHCs in the area of interest) − [(number of pixels in the area of interest) × (average background PHCs per pixel)]. Pseudo color images were generated using arbitrary color bars representing standard light intensity levels for Pluc-G-U87-MG cells (blue: lowest; red: highest). Statistical analysis was performed using Prism (GraphPad sorftware). Differences between experimental groups, at each time-point, were analyzed by paired samples multiple *t*-test, for tumor growth and animal weight. Animal survival was analyzed using Kaplan-Meier curves, applying a Log-rank (Mantel-Cox) test.

## 3. Results and discussion

### 3.1. Quality: physicochemical characterization

#### 3.1.1. Polymer quality assessment

Block co-polymer P, consisting of a rigid hydrophobic polyester block and a flexible hydrophilic PEG block, was synthesized by a polycondensation reaction. Reproducible properties of the resulting material are specified in Figure 1(B) (see results of three independent batches of co-polymer P synthesis in Supplementary Table S1). The co-polymer P has a molecular weight of around 13,000–15,000 Da (Figure 1(B)) with a narrow PDI. As determined by ^1 ^H-NMR, the P copolymer comprised a molar ratio of 60% of PEG (example on Supplementary Figure S2). The PEG content on the co-polymer molecule is a parameter of utmost importance, since it will determine the size of resulting nanoparticles. Previous studies indicated that the addition of higher PEG ratios enabled the preparation of smaller nanoparticles, which was attributed to the change on the amphiphilicity of the polymer (data not shown). Polymer molecular weight was measured by GPC (see Supplementary Figure S3(A)). In addition, NCL guidelines were applied to assess polymer quality. Chromatographic GPC performance of three different P polymer batches was practically identical, right after production and post four months storage at −20 °C (see results on Explanation S6, Supplementary material) showing that the polymer is stable during storage. Moreover, GPC results showed no traces of free PEG, confirming a complete reaction of PEG with the polyester polymer. Small peaks in the chromatogram at late times were found in the polymer as well as blank samples after long storage times which corresponded to impurities in the system.

Polymer P was also used for the synthesis of the co-polymer 2 P (Figure 1(C)), by covalently attaching the peptide targeting sequence, as described in Section 2. Similar to polymer P, to confirm that a polymer 2 P batch was appropriate, it had to fulfill the specifications detailed in Figure 1(D) (see results of 2 P co-polymer characterization in Supplementary Table S2); in particular two parameters were determined by ^1 ^H-NMR: polyester peaks and peptide peaks (see an example of ^1 ^H-NMR spectra on Supplementary Figure S4). These properties were maintained for at least six weeks at −20 °C, thus confirming the stability of this frozen polymer (Explanation S7, Supplementary material). In addition, no new peaks were detected.

As indicated above, once the synthesis procedures of both polymers were completely controlled, a scaling up of the synthesis of the polymer P was performed, since it is needed in a higher amount than the 2 P polymer. The same specifications were tested to confirm that the scale up did not produce different products; thus reconfirming the robustness of the preparation procedure and the quality of the obtained polymer batches. Supplementary Figure S3(A) shows as an example of three polymer P batches analyzed which were found to showe an almost identical chromatographic profile, that fulfills the specifications related to the molecular weight.

#### 3.1.2. Nanoparticle quality assessment

Block co-polymers P and 2 P were used to formulate PTX-loaded nanoparticles by nanoprecipitation. The use of this family of block co-polymers is advantageous for the encapsulation of hydrophobic drugs, which cannot be directly administered, such as PTX. The amphiphilic behavior of the polymer enables its structuration with the hydrophobic blocks at the inner part of the nanoparticle, entrapping the drug (core), and the hydrophilic blocks as a shell, protecting the drug while in contact with the aqueous dispersant. The use of amphiphilic polymers for the nanoencapsulation of hydrophobic drugs has been widely reported in the literature (Letchford & Burt, 2007; Nicolas et al., 2013; Zhang et al., 2014). In addition, the use of an active targeting moiety, the Seq12 peptide in the present work, has been also recommended to direct the drug therapeutic effect specifically to the required site of action (Letchford & Burt, 2007; Zhang et al., 2014; Nicolas et al., 2013); in this case, to the BBB.

Multiple independent small batches (20 mg initial polymer P) and large batches (800 mg initial polymer P) were synthesized following a modified nanoprecipitation method (Fessi et al., 1989) in order to define the quality specification criteria, according to NCL guidelines. Supplementary Tables S3 and S4 show exemplary values from the preparation of three independent small and large batches, respectively, that demonstrate reproducibility as well as the scalability in nanoparticle preparation process.

##### 3.1.2.1. Nanoparticle physicochemical characterization

As shown in Table 1, nanoparticles should have sizes between 100–120 nm and a low polydispersity index, small enough to avoid embolization and a slightly negative surface charge at neutral pH, to reduce protein aggregation and nanoparticle opsonization, thus increasing blood circulation time and enabling their arrival to the BBB (Xiao et al., 2011; Dobrovolskaia & McNeil, 2013; Lv et al., 2013). The PEG content of nanoparticles must be controlled at around 45%, since higher deviations from this value result in significant changes in size.

**Table 1.** Nanoparticle quality specification criteria.

Parameter	Specifications
Hydrodynamic diameter (nm) by DLS	100–150
PDI by DLS	<0.25
Surface charge (mV) by electrophoretic mobility	−7 to −3
PEG content (%) by H.RMN	42–46
Drug content by UPLC	1.7–2.2
Entrapment efficiency (%) by UPLC	>90 wt%

Although surface charge was not high enough to define nanoparticle electrostatic stability (Fonseca et al., 2002), the stability of the nanoparticles was confirmed by periodic evaluation of physicochemical parameters during storage (Explanation S8, Supplementary material). Nanoparticle stability can be attributed to the amphiphilic nature of the polymer which favors the formation of nanosphere structures (Zhu, 2013).

**Figure 2.**
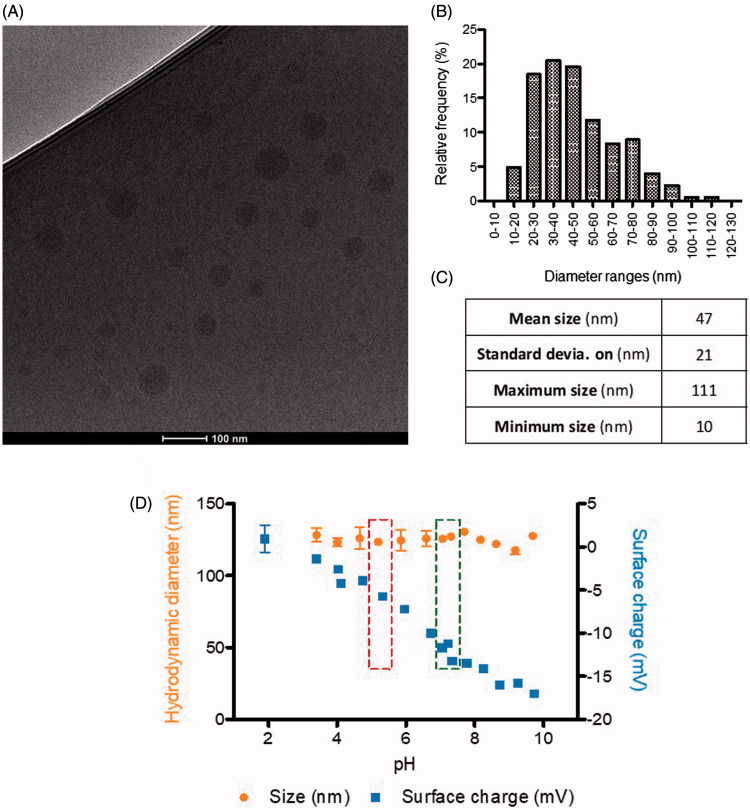
Physicochemical characterization of nanoparticles size, surface charge, and morphology: (A) CryoTEM micrography; –(B) Histogram of nanoparticles size distribution, by CryoTEM; –(C) Statistical analysis of sizes by CryoTEM (*n* = 704 nanoparticles analyzed, from different images and samples); and –(D) Hydrodynamic diameter (nm) and surface charge (mV) of nanoparticles, by DLS, as a function of pH (Left box, storage pH; right box, physiological blood pH).

Nanoparticle size and shape were further characterized by Cryo transmission electron microscopy (TEM) image analysis. As expected, nanoparticles showed smaller sizes than those obtained from DLS measurements and monomodal size distributions with mean size around 47 nm (Figure 2(B,C). In comparison with nanoparticles produced for previous studies, current nanoparticles were smaller, an advantageous trait in terms of BBB crossing and avoidance of detection by the reticuloendothelial system (RES) (Dobrovolskaia & McNeil, 2013; Tosi & Bortot, 2013). Nanoparticles had spherical morphology and smooth surface, as determined by CryoTEM (Figure 2(A)), as it had been predicted for nanosystems formed by this polymer.

It is worth remarking that, although drug content seems low (between 1.7 and 2.2%), entrapment efficiency is high; higher than 90 wt% in all synthesized NPs batches, thus indicating only less than 10 wt% of free (non-encapsulated) drug (Table S3).

Concerning drug release, as reported in Explanation S8 of Supplementary material, it is slow (only 25% released drug after 24 h) and more sustained in the presence of proteins (15% released drug after 24 h). Therefore, in blood media, it is not expected to find a PTX release, since the blood half-life is lower than 24 h, and the release would be much sustained due to the presence of proteins.

##### 3.1.2.2. Stability determination

The short-term stability of nanoparticles under physiological conditions was first studied as a function of the pH, since the pH of the different physiological compartments might be significantly different. The hydrodynamic diameter and surface charge of nanoparticles encapsulating PTX (PTX-NP) as a function of the pH are shown in Figure 2(D). Nanoparticles size was fairly constant, around 125 nm, within the pH range from 2 to 10. However, as expected, surface charge decreased notably from 0 to −18 mV with increasing pH values.

Nanoparticles stored at the pH of the dispersion media (water +10 wt% sucrose), around 4.5–5.5 , had sizes around 125 nm and surface charges around −5 mV. At the physiological pH of the blood (7.4), nanoparticles were more anionic (−18 mV), which is advantageous for the parenteral administration, since slightly negatively charged surfaces result in very low protein binding (Toti et al., 2010; Dobrovolskaia & McNeil, 2013). In addition, nanoparticles are expected to penetrate cells through the endocytic route where they are exposed to a lower pH (Panyam & Labhasetwar, 2012; Dobrovolskaia & McNeil, 2013). The protonation of the carboxylic groups of the polymer could contribute to endosomal escape of the nanoparticles due to the expected increase of the nanoparticle surface charge when pH decreases (Lv et al., 2013), making it available for therapeutic activation.

Long-term stability of nanoparticles was influenced by storage temperature and time, dispersant composition, freezing and thawing temperature, and nanoparticle concentration (see Explanation S9, Supplementary material). From these results (Explanation S9, Supplementary material), while nanoparticles were reasonably stable for at least 30 days at 4 °C, when dispersed in water +10 wt% sucrose, the best long-term storage condition was −80 °C in the same dispersant. Under these conditions nanoparticles maintained their physicochemical properties, for at least, 50 days. Conversely, freeze-drying had a negative effect on these nanoparticles.

### 3.2. Safety

Prior to *in vitro* safety, potential bacterial endotoxin contamination was discarded in all nanoparticle dispersions to <0.5 EU/mL, as determined by the FDA criteria (data not shown). In the present work, *in vitro* cytotoxicity and hemocompatibility have been studied as first indications of NPs safety. However, the authors consider that further toxicity experiments (e.g. immunological toxicity) would be required when starting preclinical regulatory studies.

#### *3.2.1.* In vitro *evaluation of nanoparticle cytotoxicity*

**Figure 3.**
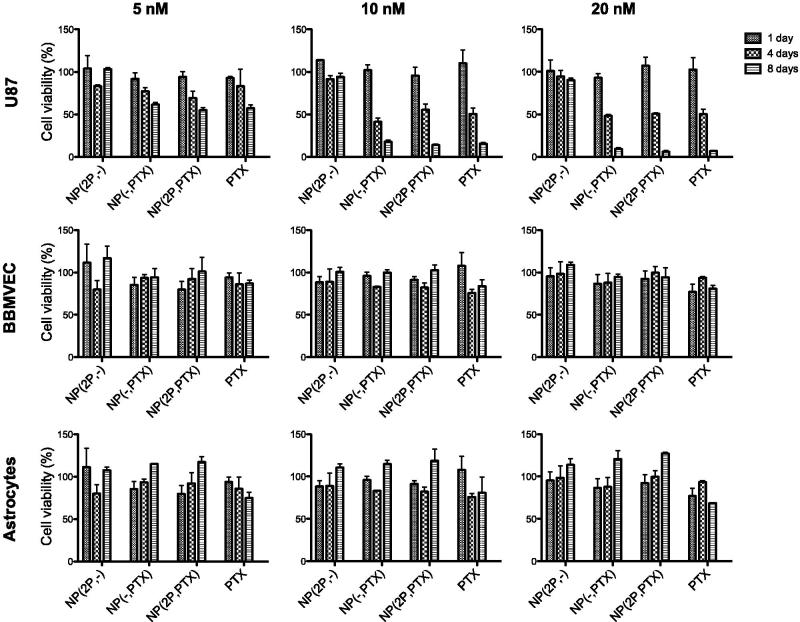
Viability (in %) of U87-MG; BBMVEC, and astrocytes cells after one, four and 8 days of samples incubation, at 5, 10 and 20 nM PTX concentration, respectively. NP(2 P,-): 2 P-decorated, non-loaded; NP(PTX): non-decorated, PTX-loaded; NP(2 P,PTX): 2 P-decorated, PTX-loaded.

As shown in Figure 3, non-loaded nanoparticles were nontoxic at all the tested concentrations, for any of the cell types, independently of the proliferation state of the cells. Decoration state of the nanoparticles seemed not to have an effect, since particles with and without the targeting moiety seem to be equally toxic to U87 cells (Figure 3). Nanoparticles did appear to have an effect sequestering PTX from culture medium and as shown in Figure 3, PTX in nanoparticles appears to be less toxic when are left free in the medium at shorter times. However, the results also showed that this nanoparticles can be used as sustained release platform. After seven days, the induced cytotoxicity of the loaded nanoparticles in dividing cells, was equivalent to that of free PTX. In addition, it is worth remarking that any type of nanoparticles tested produced no cytotoxicity in non-dividing (healthy) cells, thus ensuring their safety in normal surrounding tissues. Since central nervous system tissues are composed of non-dividing cells, although these NPs are only targeted to the BBB, if they are able to penetrate it, they are expected to produce significantly lower side-effects than the free drug, since they would not affect non-dividing cells.

#### 3.2.2. Hemocompatibility assessment

Since these NPs are intended to be administered intravenously, it was decided to perform a hemolysis test to demonstrate that erythrocytes were not lysed by synthesized NPs. Hemolytic *in vitro* tests performed using 60 and 6 mg/mL nanoparticle concentrations in mouse blood showed hemolysis levels lower than 25%, (Table S5, Supplementary material), indicating that nanoparticles are safe and can be accepted for parenteral administration; as suggested by previous studies (Amin & Dannenfelser, 2006; Dobrovolskaia & McNeil, 2013).

### 3.3. Efficacy

#### 3.3.1. Efficacy of nanoparticle BBB penetration

**Figure 4.**
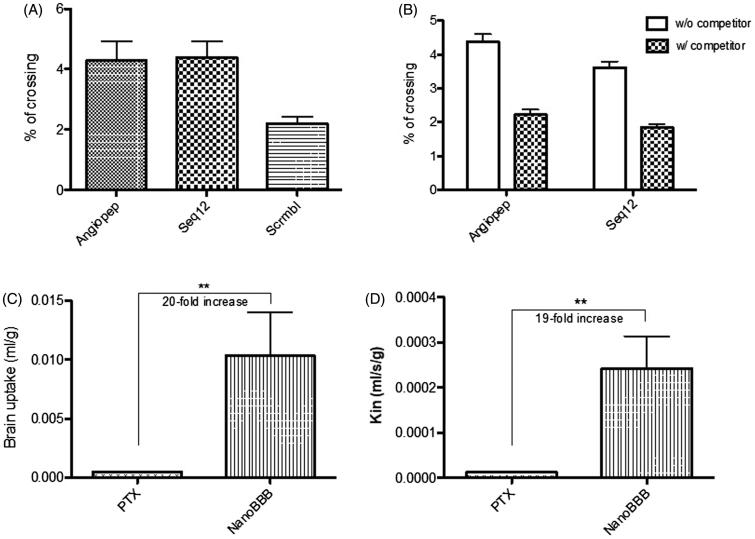
–(A) BBB crossing percentage of Angiopep-2 (ANG), Seq12, and SCRB (random; scrambled) peptide sequences and –(B) BBB crossing percentage of fluorescently-labeled Angiopep-2 and Seq12 in the absence (W/O competitor) and presence (W/ competitor) of an unlabeled competitor; –(C) Brain uptake and D - *K_in_* parameter of free PTX (PTX) and PTX-NPs (Nano-BBB). **p < .01.

Angiopep-2 is a well know peptide, recognized to cross the BBB; mainly due to its interaction with the LRP-1 receptor, overexpressed in the BBB (Régina et al., 2009); although it can also use other receptors if the LRP-1 is less expressed (Kim et al., 2016). Seq12 peptide is a Sagetis proprietary peptide, specifically designed to interact with a specific domain of the LRP receptors family, as demonstrated by a computational docking study performed to specifically design this peptide (Borrós et al., 2014). Therefore, this peptide would target not only to the BBB, where LRP receptors are overexpressed, and also in the glioma cells, where LRP-1 is also seen to be overexpressed (Borrós et al., 2014). The capacity of Seq12 (Borrós et al., 2014) peptide to cross the BBB was evaluated, in comparison to Angiopep-2 (ANG) (Régina et al., 2009) peptide, to determine whether it was adequate to target the BBB. As shown in Figure 4(A), Seq12, crossed the BBB with an efficiency equivalent to that of ANG (4.28 ± 0.64 vs. 4.39 ± 0.54% respectively) (Demeule et al., 2008; Bertrand et al., 2011; Oller-Salvia et al., 2016).

In addition, competition assays showed that BBB crossing of ANG and Seq12 peptides was similarly reduced by the corresponded unlabeled peptides (4.38 ± 0.22 vs. 2.22 ± 0.15% and 3.62 ± 0.18 vs. 1.84 ± 0.09%, respectively (Figure 4B), indicating that that receptors of the LRP family may probably be involved in the BBB crossing of the Seq12 peptide (Demeule et al., 2008; Bertrand et al., 2011). This result confirmed Seq12 as a good candidate to be used as BBB shuttle. As compared with other peptides, discovered some years before, Seq12 peptide shows an increased efficiency, equivalent to that of ANG (Demeule et al., 2008; Bertrand et al., 2011; Oller-Salvia et al., 2016).

Next, we evaluated the capacity of PTX-loaded Seq12-decorated nanoparticles to cross the BBB in an *in vivo* setting, performing *in situ* brain perfusion studies. It is worth taking into account that, although during glioma illness the BBB is impaired, these experiments were performed with healthy animals, in a more restrictive condition. In addition, the PTX is a substrate of the P-gp, which is intact during the disease (Régina et al., 2009). Plots in Figure 4(C,D) show that Seq12-decorated PTX-NPs were significantly better (approx. 20×; with *p* < .01) at crossing the BBB than free PTX, according to the two parameters. Therefore, this study confirms the Seq12 capacity to cross the BBB (see Supplementary Figure S5 where it is demonstrated that Seq12 peptide and not only the encapsulation of the drug enable the PTX BBB crossing).

In addition, it would appear that our Seq12-decorated PTX-NPs have a better brain perfusion capacity than other reported formulations. For example, Régina et al. (2009) reported a five-fold *K_in_* increase of their ANG1005 formulation, as compared with free PTX in mice perfusion studies.

It is worth remarking that, although brain perfusion studies are usually performed with healthy mice (Régina et al., 2009), as in the current article, the development of glioblastoma multiforme ends up with the impairment of the BBB, thus facilitating the crossing of exogenous components. Nevertheless, the results found with healthy mice confirm the capacity of the Seq12 peptide to efficiently cross the BBB and not being expulsed by the P-gp e-flux pump (Figure 4(C,D). 

#### *3.3.2.* In vivo *therapeutic efficacy*

The *in vivo* study was performed to demonstrate the efficacy of the designed nanoparticles (PTX-loaded and Seq12 targeting) to treat GBM. Three parameters: tumor growth, animals weight and survival were evaluated to determine the therapeutic efficacy of the Seq12-decorated PTX-NP formulation.

##### 3.3.2.1. NPs reduced tumor growth

**Figure 5.**
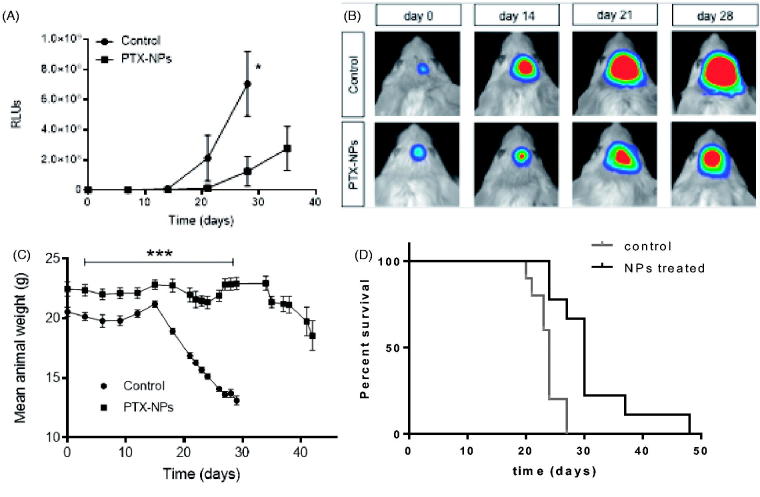
–(A) Growth of luciferase expressing U87-MG tumors in live mice intravenously treated with either PTX-loaded NP or empty NPs, (*p < .05; *n* = 10); –(B) Bioluminescence images of tumors showing Pluc-G-U87-MG cells activity at days 7, 14, 21, and 28 from representative mice in each group. Pseudo-color images are superimposed on black and white dorsal images of the corresponding animal. Light intensity is represented by an arbitrary color scale (center-bright = high, darkborders = low). –(C) Mean animals weight as a function of treatment time (***p < .001; *n* = 10). –(D) Kaplan-Meyer curves summarizing mice survival. Log-rank (Mantel-Cox) test; (*p* = .001; *n* = 10). Treatment schedule: injection every three days up to day 21.

A luciferase expressing U87 tumor model in SCID mice was used to monitor *in vivo* tumor response to PTX-NP treatment. Plots of total light events (PHCs) vs. time as well as representative images of experimental animals, showed (Figure 5(A,B)  that tumors in control animals (empty NPs) grew faster compared with those in the NP-PTX treated group. The progression of the tumor is significantly delayed (*p* < .05) when PTX was encapsulated in NP, after 28 days of treatment.

##### 3.3.2.2. NPs decreased weight loss

Plots of weight loss, an indication of overall animal health, was evaluated for the same animals (Figure 5(C)). They showed that PTX-NP treated animals were significantly healthier throughout the experiment, comparing with the weight of control animals, been approx. 30% lower by day 21. It is also remarkable that, after treatment time (21 days), NPs-treated animals recovered weight.

##### 3.3.2.3. NPs increased animals’ survival

Further support for the antitumor efficacy of PTX-NPs was provided by Kaplan-Meier analysis of animal survival in the experiment showing (Figure 5(D)) that the PTX-NP group had a significant 50% increase in life span and a 30 days median survival while 100% of those treated with empty NPs died by day 29.

After presenting all these results, it is clear that PTX has an efficient therapeutic effect on mice. Experiments in humans demonstrated the complete inability of free PTX to cross the BBB (Glantz et al., 1995), a reason for why it is not the standard of care for glioblastoma multiforme. Therefore, although in the present study PTX was selected as the active principle, it could not be used for a human therapy without being encapsulated. The current standard of care, temozolomide (TMZ) (and radiotherapy following surgical intervention), was not tested in this work because the effect of this drug is dependent on tumor genetic expression (Stupp et al., 2009; Chamberlain, 2010). Patients with high levels of methylation in the promoter of the alkylguanine alkyl transferase (AGT) gene showed tumor sensitivity to TMZ. However, it was previously demonstrated that TMZ is not a curative treatment in most patients, since relapses are frequent after this treatment and it creates secondary resistance together with metastases development (Stupp et al., 2009; Chamberlain, 2010). The tumor model used in the current study, U87-MG glioblastoma cells, was selected because it is the most commonly used tumor model in previous studies. Nevertheless, these cells have very high levels of AGT methylation, thus showing high sensibility to TMZ (Kanzawa et al., 2003, 2004). Therefore, it would be expected that a high efficacy of TMZ could be observed in this animal model but it would not represent a model of TMZ behavior in patients. So it was ruled out as model of current standard of care in the present study conditions.

Concerning our formulation, within the *in vivo* studies, it has been demonstrated its ability to cross an integer BBB (with the *in situ* brain perfusion study) and its therapeutic efficacy. The treatment schedule for the *in vivo* tumor growth experiments provides PTX treatment until day 21. Once the treatment is finished, another treatment round could be applied to these animals to increase treatment efficacy.

In addition, compared with other studies, our nanoparticles demonstrated an enhancement of life survival. The main potential competitor is Angiochem, who achieved 15% of mice ILS using their product ANG1005, a nanocomplex composed of PTX conjugated with a brain peptide vector (Demeule et al., 2008; Régina et al., 2009; Bertrand et al., 2011). Both experiments are fairly comparable in terms of cells used (U87-MG), similar amount of cells implanted and intracranial cell implantation. Even though in the present study less than half the dose of PTX was administered to mice, it was achieved a 50% ILS. In addition, in the study of (Demeule et al. (2008) and Régina et al. (2009) most experiments were performed with tumor xenografts (not intracranial tumors), where the difficulties of crossing the BBB do not exist and in the only study with intracranial tumors. Therefore, it is reasonable to assume that in the current study, the delivery of PTX in tumor cells is better than in previous studies, due to the use of PTX-NPs and this formulation accomplished with the efficacy principle of the NCL guidelines.

Considering that ANG1005 has reached clinical trials demonstrating similar results to those presented in the current work, the next step of the present study would be a regulatory preclinical study in animal models to further reach to clinical studies if the results are promising. It is worth noting the importance of the deep characterization using the assay protocol cascade proposed in this article. This characterization enables the selection of the best candidate to start the *in vivo* efficacy studies in animal models, performed here; which is more reliable than the use of a non-deeply characterized nanoparticles.

## 4. Conclusion

Polymeric nanoparticles for GBM therapy have been designed, synthetized, and extensively characterized with the NCL assay cascade protocol designed to evaluate the physicochemical characteristics (quality) to biocompatibility (safety) and *in vivo* therapeutic performance (efficacy).

This strategy facilitated the development of a novel nanomedicine that is physicochemically well characterized, safe for cells in culture, and for physiological delivery and effective for therapy. Thus, the developed NPs showed reduced cell toxicity in *in vitro* assays and when decorated with Seq12 peptide they improved PTX penetration through the BBB. Finally, PTX-loaded NPs were effective at treating a U87 glioma model in live mice, reducing tumor growth and significantly increasing animal survival. For all these reasons, it can be concluded that our formulation has been widely characterized using the NCL assay cascade protocol proposed here and confirmed as an effective nanosystem in animal models, with therapeutic effects notably higher than current and other under investigation glioblastoma treatments. Therefore, thanks to the whole characterization study of the quality, safety, and efficacy of the system and also to the promising results obtained, the nanoparticles developed on this experimentation are ready to regulatory preclinical evaluation to assess the dosage and therapeutic regime of the proposed treatment. We hypothesize that the present NPs will probably be administered in combination with current treatments (TMZ and radiotherapy), to increase the overall efficacy in clinical studies.

## Supplementary Material

IDRD_Borr_s_et_al_Supplemental_Content.pdf

## References

[CIT0001] Alieva M, Bagó JR, Aguilar E, et al (2012). Glioblastoma therapy with cytotoxic mesenchymal stromal cells optimized by bioluminescence imaging of tumor and therapeutic cell response. PLoS ONE 7:1–11. 10.1371/journal.pone.0035148 PMC3328467 22529983

[CIT0002] Alyautdin R, Khalin I, Nafeeza MI, et al (2014). Nanoscale drug delivery systems and the blood-brain barrier. Int J Nanomedicine 9:795–811. 24550672 10.2147/IJN.S52236 PMC3926460

[CIT0003] Amin K, Dannenfelser RM. (2006). In vitro hemolysis: guidance for pharmaceutical scientist. J Pharm Sci Exp Pharmacol 95:1173–6. 10.1002/jps.20627 16639718

[CIT0004] Bagó JR, Alieva M, Soler C, et al (2013). Endothelial differentiation of adipose tissue-derived mesenchymal stromal cells in glioma tumors: implications for cell-based therapy. Mol Ther 21:1758–66. 23760448 10.1038/mt.2013.145 PMC3776633

[CIT0005] Bazile DV. (2014). Nanotechnologies in drug delivery - an industrial perspective. J Drug Deliv Sci Technol 24:12–21.

[CIT0006] Béduneau A, F, Hindré A, Clavreul JC, et al (2008). Brain targeting using novel lipid nanovectors. J Control Release 126:44–9. 18055056 10.1016/j.jconrel.2007.11.001

[CIT0007] Bertrand Y, Currie JC, Poirier J, et al (2011). Influence of glioma tumour microenvironment on the transport of ANG1005 via low-density lipoprotein receptor-related protein 1. Br J Cancer 105:1697–707. 22027709 10.1038/bjc.2011.427 PMC3242593

[CIT0008] Bickel U. (2005). How to measure drug transport across the blood-brain barrier. Neurotherapeutics 2:15–26. 10.1602/neurorx.2.1.15 PMC539317 15717054

[CIT0009] Boje KMK. (2002). Unit 7.4: in vivo measurement of blood-brain barrier permeability In: Skolnick P, ed. Current protocols in neurosciences. New York: John Wiley & Sons, 7.19.1–7.19.39.

[CIT0010] Borrós GS, Rivero MFX, Cascante CA. (2014). Polypeptides for blood-brain barrier transport. Patent publication number: WO 2014076655 A1.

[CIT0011] Chaichana KL, Jusue-Torres I, Navarro-Ramirez R, et al (2014). Establishing percent resection and residual volume thresholds affecting survival and recurrence for patients with newly diagnosed intracranial glioblastoma. Neuro-Oncology 16:113–22. 24285550 10.1093/neuonc/not137 PMC3870832

[CIT0012] Chaichana KL, Zadnik P, Weingart JD, et al (2013). Multiple resections for patients with glioblastoma: prolonging survival. J Neurosurg 118:812–20. 23082884 10.3171/2012.9.JNS1277 PMC3700339

[CIT0013] Chamberlain MC. (2010). Temozolomide: therapeutic limitations in the treatment of adult high-grade gliomas. Expert Rev Neurother 10:1537–44. 20925470 10.1586/ern.10.32

[CIT0014] Chamberlain MC, Kormanik P. (1995). Salvage chemotherapy with paclitaxel for recurrent primary brain tumors. J Clin Oncol 13: 2066–71. 7636549 10.1200/JCO.1995.13.8.2066

[CIT0015] Crown J, O’Leary M, Ooi WS. (2004). Docetaxel and paclitaxel in the treatment of breast cancer: a review of clinical experience. Oncologist 9: 24–32. 15161988 10.1634/theoncologist.9-suppl_2-24

[CIT0016] Demeule M, Currie JC, Bertrand Y, et al (2008). Involvement of the low-density lipoprotein receptor-related protein in the transcytosis of the brain delivery vector angiopep-2. J Neurochem 106: 1534–44. 18489712 10.1111/j.1471-4159.2008.05492.x

[CIT0017] Dobrovolskaia MA, Clogston JD, Neun BW, et al (2008). Method for analysis of nanoparticle hemolytic properties in vitro. Nano Lett 8:2180–7. 18605701 10.1021/nl0805615 PMC2613576

[CIT0018] Dobrovolskaia MA, McNeil SE. (2007). Immunological properties of engineered nanomaterials. Nat Nanotechnol 2:469–78. 18654343 10.1038/nnano.2007.223

[CIT0019] Dobrovolskaia MA, McNeil SE, eds. (2013). Handbook of Immunological Properties of Engineered Nanoparticles. 2nd ed Singapore: World Scientific Publishing.

[CIT0020] Duncan R, Gaspar R. (2011). Nanomedicine(s) under the microscope. Mol Pharm 8:2101–41. 21974749 10.1021/mp200394t

[CIT0021] Fessi H, Puisieux F, Devissaguet JP, et al (1989). Nanocapsule formation by interfacial polymer deposition following solvent displacement. Int J Pharm 55:1–4.

[CIT0022] Fonseca C, Simoes S, Gaspar R. (2002). Paclitaxel-loaded PLGA nanoparticles: preparation, physicochemical characterization and in vitro anti-tumoral activity. J Control Release 83:273–86. 12363453 10.1016/s0168-3659(02)00212-2

[CIT0023] Gaudin A, Andrieux K, Couvreur P. (2015). Nanomedicines and stroke: toward translational research. J Drug Deliv Sci Technol 30:278–99.

[CIT0024] Ge Y, Li S, Wang S, Moore R, Eds. (2014). Nanomedicine. Otawa: Springer.

[CIT0025] Glantz MJ, Choy H, Kearns CM, et al (1995). Paclitaxel disposition in plasma and central nervous systems of humans and rats with brain tumors. J Natl Cancer Inst 87: 1077–81. 7616600 10.1093/jnci/87.14.1077

[CIT0026] Gref R, Domb A, Quellec P, et al (1995). The controlled intravenous delivery of drugs using PEG-coated sterically stabilized nanospheres. Adv Drug Deliv Rev 16:215–33. 25170183 10.1016/0169-409X(95)00026-4 PMC4144462

[CIT0027] Hall JB, Dobrovolskaia MA, Patri AK, McNeil SE. (2007). Characterization of nanoparticles for therapeutics. Nanomedicine 2:789–803. 18095846 10.2217/17435889.2.6.789

[CIT0028] Kabanov AV, Gendelman HE. (2007). Nanomedicine in the diagnosis and therapy of neurodegenerative disorders. Prog Polym Sci (Oxford) 32:1054–82. 10.1016/j.progpolymsci.2007.05.014 PMC2838200 20234846

[CIT0029] Kanzawa T, Germano IM, Komata T, et al (2004). Role of autophagy in temozolomide-induced cytotoxicity for malignant glioma cells. Cell Death Differ 11:448–57. 14713959 10.1038/sj.cdd.4401359

[CIT0030] Kanzawa T, Germano IM, Kondo Y, et al (2003). Inhibition of telomerase activity in malignant glioma cells correlates with their sensitivity to temozolomide. Br J Cancer 89:922–9. 12942127 10.1038/sj.bjc.6601193 PMC2394478

[CIT0031] Kim JA, Casalini T, Brambilla D, Leroux JC. (2016). Presumed LRP1-targeting transport peptide delivers β-secretase inhibitor to neurons in vitro with limited efficiency. Sci Rep 6:34297. 27682851 10.1038/srep34297 PMC5041153

[CIT0032] Kreuter J. (2014). Drug delivery to the central nervous system by polymeric nanoparticles: what do we know? Adv Drug Deliv Rev 71: 2–14. 23981489 10.1016/j.addr.2013.08.008

[CIT0033] Letchford K, Burt H. (2007). A review of the formation and classification of amphiphilic block copolymer nanoparticulate structures: micelles, nanospheres, nanocapsules and polymersomes. Eur J Pharm Biopharm 65:259–69. 17196803 10.1016/j.ejpb.2006.11.009

[CIT0034] Lv S, Li M, Tang Z, et al (2013). Doxorubicin-loaded amphiphilic polypeptide-based nanoparticles as an efficient drug delivery system for cancer therapy. Acta Biomater 9:9330–42. 23958784 10.1016/j.actbio.2013.08.015

[CIT0035] McGirt MJ, Mukherjee D, Chaichana KL, et al (2009a). Association of surgically acquired motor and language deficits on overall survival after resection of glioblastoma multiforme. Neurosurgery 65:463–9. 19687690 10.1227/01.NEU.0000349763.42238.E9

[CIT0036] McGirt MJ, Than KD, Weingart JD, et al (2009b). Gliadel (BCNU) wafer plus concomitant temozolomide therapy after primary resection of glioblastoma multiforme. J Neurosurg 110:583–8. 19046047 10.3171/2008.5.17557 PMC4856017

[CIT0037] McGrogan BT, Gilmartin B, Carney DN, McCann A. (2008). Taxanes, microtubules and chemoresistant breast cancer. Biochim Biophys Acta 1785:96–132. 18068131 10.1016/j.bbcan.2007.10.004

[CIT0038] McNeil SE. (2011). Characterization of nanoparticles intended for drug delivery. New York: Springer.

[CIT0039] Miele E, Gian Paolo S, Ermanno M, et al (2009). Albumin-bound formulation of paclitaxel (AbraxaneABI-007) in the treatment of breast cancer. Int J Nanomedicine 4:99–105. 19516888 10.2147/ijn.s3061 PMC2720743

[CIT0040] Mitragotri S, Anderson DG, , et al. (2015). Accelerating the translation of nanomaterials in biomedicine. ACS Nano 9:6644–54. 26115196 10.1021/acsnano.5b03569 PMC5227554

[CIT0041] Mura S, Couvreur P. (2012). Nanotheranostics for personalized medicine. Adv Drug Deliv Rev 64: 1394–416. 22728642 10.1016/j.addr.2012.06.006

[CIT0042] Neha B, Ganesh B, Preet K. (2013). Drug delivery to the brain using polymeric nanoparticles: a review. Int J Pharma Life Sci 2:107–32.

[CIT0043] Nicolas J, Mura S, Brambilla D, et al (2013). Design, functionalization strategies and biomedical applications of targeted biodegradable/biocompatible polymer-based nanocarriers for drug delivery. Chem Soc Rev 42:1147–235. 23238558 10.1039/c2cs35265f

[CIT0044] Oller-Salvia B, Sánchez-Navarro M, Giralt E, Teixidó M. (2016). Blood-brain barrier shuttle peptides: an emerging paradigm for brain delivery. Chem Soc Rev 45:4690–707. 27188322 10.1039/c6cs00076b

[CIT0045] Ostrom QT, Gittleman H, Liao P, et al (2014). CBTRUS statistical report: primary brain and central nervous system tumors diagnosed in the United States in 2007-2011. Neuro Oncol 16:iv1–iv63. 25304271 10.1093/neuonc/nou223 PMC4193675

[CIT0046] Panyam J, Labhasetwar V. (2012). Biodegradable nanoparticles for drug and gene delivery to cells and tissue. Adv Drug Deliv Rev 64 : 61–71. 10.1016/s0169-409x(02)00228-4 12628320

[CIT0047] Pardridge WM. (2012). Drug transport across the blood–brain barrier. J Cereb Blood Flow Metab 32:1959–72. 22929442 10.1038/jcbfm.2012.126 PMC3494002

[CIT0048] Pinto Reis C, Neufeld RJ, Ribeiro AJ, Veiga F. (2006). Nanoencapsulation I. Methods for preparation of drug-loaded polymeric nanoparticles. Nanomedicine 2: 8–21. 17292111 10.1016/j.nano.2005.12.003

[CIT0049] Régina A, Demeule M, Ché C, et al (2009). Antitumour activity of ANG1005, a conjugate between paclitaxel and the new brain delivery vector angiopep-2. Br J Pharmacol 155:185–97. 10.1038/bjp.2008.260 PMC2538693 18574456

[CIT0050] Stupp R, Hegi ME, Mason WP, et al (2009). Effects of radiotherapy with concomitant and adjuvant temozolomide versus radiotherapy alone on survival in glioblastoma in a randomised phase III study: 5-year analysis of the EORTC-NCIC trial. Lancet Oncolo 10: 459–66. 10.1016/S1470-2045(09)70025-7 19269895

[CIT0051] Tosi G, Bortot B. (2013). Potential use of polymeric nanoparticles for drug delivery across the blood-brain barrier. CurrMed Chem 20:2212–25. 10.2174/0929867311320170006 23458620

[CIT0052] Toti US, Guru BR, Grill AE, Panyam J. (2010). Interfacial activity assisted surface functionalization: a novel approach to incorporate maleimide functional groups and cRGD peptide on polymeric nanoparticles for targeted drug delivery. Mol Pharmaceutics 7: 1108–17. 10.1021/mp900284c PMC2914136 20527782

[CIT0053] Vauthier C, Bouchemal K. (2009). Methods for the preparation and manufacture of polymeric nanoparticles. Pharm Res 26:1025–58. 19107579 10.1007/s11095-008-9800-3

[CIT0054] Venditto VJ, Szoka FC. (2013). Cancer nanomedicines: so many papers and so few drugs!. Adv Drug Deliv Rev 65:80–8. 23036224 10.1016/j.addr.2012.09.038 PMC3565003

[CIT0055] Venkatraman S. (2014). Has nanomedicine lived up to its promise? Nanotechnology 25: 372501. 25148691 10.1088/0957-4484/25/37/372501

[CIT0056] Xiao K, Li Y, Luo J, et al (2011). The effect of surface charge on in vivo biodistribution of PEG-oligocholic acid based micellar nanoparticles. Biomaterials 32:3435–46. 21295849 10.1016/j.biomaterials.2011.01.021 PMC3055170

[CIT0057] Zhang X. (2011). Glioblastoma multiforme: molecular characterization and current treatment strategy (Review). Exp Ther Med 3:9–14. 22969836 10.3892/etm.2011.367 PMC3438851

[CIT0058] Zhang M, Herion TW, Timke C, et al (2011). Trimodal glioblastoma treatment consisting of concurrent radiotherapy, temozolomide, and the novel TGF-β receptor I kinase inhibitor LY2109761. Neoplasia 13:537–49. 21677877 10.1593/neo.11258 PMC3114247

[CIT0059] Zhang S, Wu Y, He B, et al (2014). Biodegradable polymeric nanoparticles based on amphiphilic principle: construction and application in drug delivery. Sci China Chem 57:461–75.

[CIT0060] Zhu Z. (2013). Effects of amphiphilic diblock copolymer on drug nanoparticle formation and stability. Biomaterials 34: 10238–48. 24070569 10.1016/j.biomaterials.2013.09.015 PMC3830127

[CIT0061] Zlokovic BV. (2008). The blood-brain barrier in health and chronic neurodegenerative disorders. Neuron 57: 178–201. 18215617 10.1016/j.neuron.2008.01.003

