# Supplementary material for: Application of an assay Cascade methodology for a deep preclinical characterization of polymeric nanoparticles as a treatment for gliomas
Source: Drug Deliv. 2018 Feb 7;25(1):472–83. doi: 10.1080/10717544.2018.1436099 (PMC6058495; doi:10.1080/10717544.2018.1436099)
Supplement: IDRD_Borr_s_et_al_Supplemental_Content.pdf [file IDRD_A_1436099_SM3357.pdf]

Standardization of an assay cascade methodology for a deep preclinical characterization of polymeric nanoparticles as a treatment for gliomas

C. Fornaguera, M.A. Lázaro, P. Brugada-Vilà, I. Porcar, M. Guerra-Rebollo, C. Garrido, N. Rubió, J. Blanco, A. Cascante, S. Borrós

Supplementary information

Index

Explanations:
Explanation S1: Characterization of synthesized polymers.
Explanation S2: Characterization of polymeric nanoparticles.
Explanation S3: Characterization of the BBB *in vitro* model.
Explanation S4: Final point criteria for animal sacrifice.
Explanation S5: Lentiviral plasmids preparation and culture of U87-MG cells for in vivo tumor implantation
Explanation S6: Study of the stability of polymer P.
Explanation S7: Study of the stability of polymer 2P.
Explanation S8: PTX release studies.
Explanation S9: Nanoparticles long-term stability study.

Figures:
Figure S1: Schematic representation of the in vitro BBB models. BBMVECs are cultured on the collagen coated insert surface and astrocytes are grown below, on the bottom of the multi-well plate.
Figure S2: ^{1}H-NMR of an example batch of the P co-polymer.
Figure S3: GPC of three big batches of the polymer P, at a) initial preparation time (t=0) and b) after 4 months stored at -20ºC.
Figure S4: ^{1}H-NMR of an example batch of the polymer 2P, at a) initial preparation time (t=0) and b) after 6 weeks stored at -20ºC.
Figure S5: A - Brain uptake and B - K_{in} parameter of free PTX and PTX-NPs, functionalized with Seq12 peptide NPs(Seq12) and without any functionalization NPs(NF). **p<0.01.

Tables:

Table S1: Physicochemical characterization of three independent batches of polymer P.
Table S2: Physicochemical characterization of three independent batches of polymer 2P.
Table S3: Physicochemical characterization of three independent small batches of nanoparticles.
Table S4: Physicochemical characterization of three independent big batches of nanoparticles.
Table S5: Nanoparticle induced hemolysis (%). n=6replicates/sample. Note that PTX concentrations are much higher than in in vitro experiments because in hemolysis experiments, the expected injected concentration must be tested, and it is always higher in vivo than in vitro.

Explanation S1: Characterization of synthesized polymers

- Gel permeation chromatography (GPC)

Gel permeation chromatography (GPC) was used to determine the molecular weight of the synthesized polymers. Around 1.5 mg of the polymer and polystyrene standards (MW = 2500; 5000; 9000; 17500; 30000 Da; calibration curve) were disolved in 1 mL tetrahydrofurane. 20 µL of each sample were analyzed on a GPC KF-603 column (3 [illegible]m, 6.0x150) from SHODEX with a flow rate of 0.5 mL/min of tetrahydrofurane. The calibration curve was obtained by extrapolation from the logarithm of the molecular weight of the polystyrene standard as a function of the retention time. The Mw (weight average molecular weight), Mn (number average molecular weight) and PDI (polydispersity) were obtained by analysis of the data with Microsoft Excel software (Equations 3,4,5, respectively; where h is the Height and M is the mass obtained from retention time with the calibration curve). GPC was performed at different time-points, to determine the stability of the polymer as a function of the temperature

$$Mw = \frac{\sum h * M}{\sum h} \quad \text{(Equation 3)}$$

$$Mn = \frac{\sum h}{\sum h/M} \quad \text{(Equation 4)}$$

$$PDI = \frac{Mw}{Mn} \quad \text{(Equation 5)}$$

- ^{1}H -NMR

Proton nuclear magnetic resonance (^{1}H -NMR) was performed to determine the ratio of PEG in the polymers P, P* and 2P and nanoparticles. In brief, around 10 mg of polymer P, P* and nanoparticles were dissolved in 0.8 mL deuterated chloroform, while polymer 2P was dissolved in dimethyl sulfoxide. ^{1}H –NMR spectra was recorded with 16 scans on a Varian 400 MHz. Data were processed using the MestReNova software. The ratio between the PEG and the polyester was determined for the polymers P, 2P or nanoparticles by integrating the signal of PEG (3.64 ppm) and that of polyester (4.06 ppm), as described by Equation 1. To obtain the percentage of peptide in the polymer 2P, the signals of PEG (3.64 ppm) and tyrosine (6.60 ppm) were integrated. The number

of protons of the signal of PEG was 256 and for the signal of tyrosine was 2. Then the formula was corrected with the number of protons. (Equation 2).

$$\% \, of \, PEG = \frac{Integral \, of \, PEG}{Integral \, of \, polyester + integral \, of \, PEG} * 100 \qquad \text{(Equation 1)}$$

$$\% \, of \, peptide = \frac{Integral \, of \, tyrosine * 256}{Integral \, of \, PEG * 2} * 100 \qquad \text{(Equation 2)}$$

- <u>Acid titration</u>

Acid titration was performed to confirm the correctness of the polymer. 250mg polymer P were weighted and dissolved in 15 mL acetone and 3 drops of 1wt% phenolphthalein in ethanol. 0.1M KOH solution in methanol was added until color changed to pink. The amount of acid per gram of polymer was calculated following Equation 3.

$$\frac{Volume \, KOH \, (mL) * 0.1}{mass \, polymer \, (g)} = mmol \, acid \, / \, g \, polymer \qquad \text{(Equation 3)}$$

Explanation S2: Characterization of polymeric nanoparticles

- Hydrodynamic size and surface charge determination

Hydrodynamic diameters of nanoparticles were determined by dynamic light scattering (DLS), using a Malvern Nano SZ, equipped with a He-Ne laser (633 nm). The scattered light was detected at 173º. Nanoparticles were diluted to 0.2 – 2.2 mg/mL to avoid multiple scattering problems, using NaCl 10mM as the diluent to ensure the appropriate ionic strength for the Brownian motion. For size assessment, Stokes-Einstein equation was used, while for surface charge, the ζ potential was calculated from the electrophoretic mobility, using the Smoluchowski approximation. Hydrodynamic diameters and surface charges were also measured as a function of the pH to study the stability of nanoparticles at different pHs, using the Autotitrator MPT-2, coupled to the instrument. Results are expressed as mean standard deviation of at least 3 replicates.

- Osmolality determination

Nanoparticle dispersions osmolality was determined by freezing point depletion technique, using a Fiske 210 Micro-Sample Osmometer. Physiological osmolalities range from 275 to 300 mOm/kg.

- Cryo Transmission electron microscopy (cryoTEM)

Hard sphere diameter and morphology of nanoparticles was assessed by cryoTEM. In brief, nanoparticle dispersions were cryofixed in liquid ethane using a Vitrobot MARK III equipment. Then, cryofixed samples were visualized using the Tecnai F20 cryoTEM microscope, setting the electron voltage to 200kV (low voltage) and the temperature between -175 and -170ºC. Using a CCD Eagle camera of 4096x4096 pixels, images were taken. Image J software was used to determine sizes.

- Ultra-high performance liquid chromatography (UPLC)
    - Drug content and entrapment efficiency determination

PTX amount was quantified using UPLC methodology (Waters ACQUITY UPLC H-Class). 1-2 mg of freeze-dried, 0.22 μm filtered nanoparticle dispersions were dissolved in 1 mL methanol and analyzed. A method with internal standards of docetaxel (DCTX) was selected. A reverse phase BEH C18 column (1.7 μm 2.1 x 50mm) was used. The mobile phase consisted of a mixture of Methanol 0.1% acetic acid and water 0.1% acetic acid (65:35 v/v), at a flow rate of 0.6 mL/min. PTX and DCTXL were quantified by UV detection (λ = 227 nm, Waters TUV detector). Retention times of PTX and DCTXL were around 0.86 minutes and 1.01 minutes, respectively. The calibration curve for quantification was obtained from PTX standard solutions. PTX quantification in sample solution was obtained from interpolation in the calibration curve. The efficiency of PTX encapsulation and drug loading were calculated using equations (6) and (7), respectively.

$$\textit{Entrapment efficiency} = \frac{\textit{initial drug amount - free drug}}{\textit{initial drug amount}} \times 100 \qquad \text{(Equation 6)}$$

$$\text{Drug content} = \frac{\text{amount of drug in nanoparticles}}{\text{100mL of nanoparticles}} \times 100 \qquad \text{(Equation 7)}$$

Explanation S3: Characterization of the BBB *in vitro* model.

In order to assess the barrier integrity, our BBB model was characterized in terms of trans-endothelial electrical resistance (TEER) and permeability (Pe) of the water-soluble molecular tracer called Lucifer Yellow (LY). Moreover, the expression pattern of tight junctions' proteins was studied by immunofluorescence techniques.

TEER value reflects the resistance to the passage of small ions through the endothelial cell layer and is an accurate and sensitive measure of BBB integrity. A decrease in TEER implies an increase in permeability and a loss of barrier function. In our model, the obtained TEER values were always higher than 200 Ωcm^2 and significantly higher in comparison with the monoculture model, demonstrating the increase of tightness induced by the presence of glial cells in the culture (Figure S3.1).

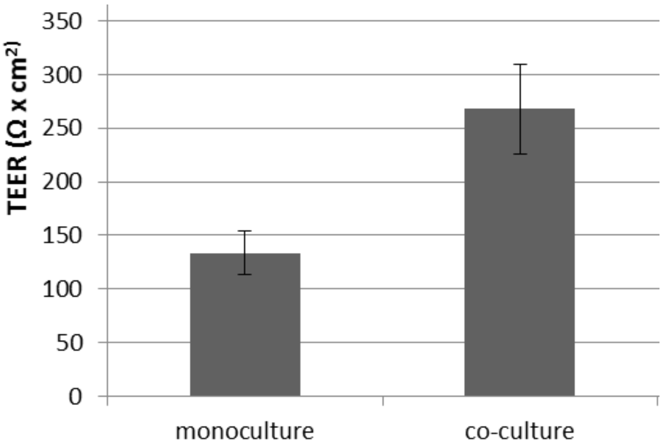

Figure S3.1: TEER values measured 6 days after BBMVEC seeding. The data are presented as mean ± SD values of at least 20 Transwell filters from 3 independent experiments.

LY is a fluorescent dye that cannot be taken up by EC *in vivo*, neither by active nor by facilitated transport; thus, the study of its permeability coefficient (Pe) is used as a marker for layer integrity and BBB quality of the *in vitro* models, together with the determination of the TEER values. As shown in Figure S3.2, the presence of glial cells in the co-culture model induced lower LY Pe values when compared to monoculture model (0.899±0.08 vs. 1.269±0.67 cm/min-1 (mean ± SD)).

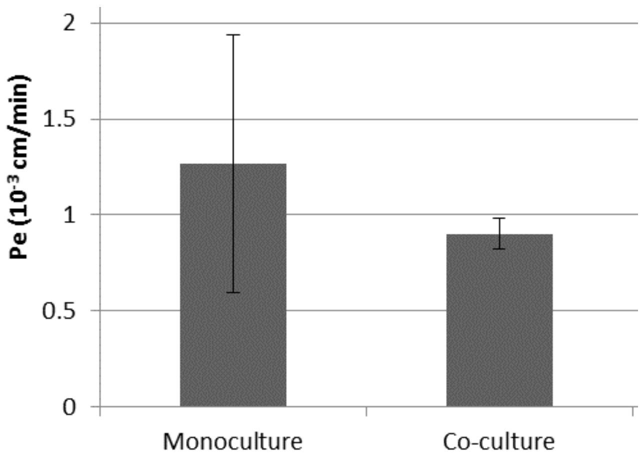

Figure S3.2: Permeability coefficient (Pe) of Lucifer Yellow. Pe values are represented as the mean ± SD.

Tight junctions (TJ) are known to be the main BBB physiological feature. TJs extend circumferentially around the endothelial cells forming a barrier to paracellular passage of small hydrophilic molecules such as sodium, hydrogen, bicarbonate, and also restricting the movements of proteins, particles like viruses and even cells across the BBB. They also play a role in endothelial cells polarization restricting the movement of membrane molecules between the apical and basolateral membrane surfaces.

In order to phenotypically assess our *in vitro* BBB model, a specific analysis of the expression pattern of tight junctions' proteins, such as ZO-1, Claudin-5 and Occludin, was carried out using immunostainning techniques. A monoculture of BBMVECs was used as a control. As shown in Figure S3.3, the expression pattern of these three proteins was increased when BBMVECs were co-cultured with glial cells.

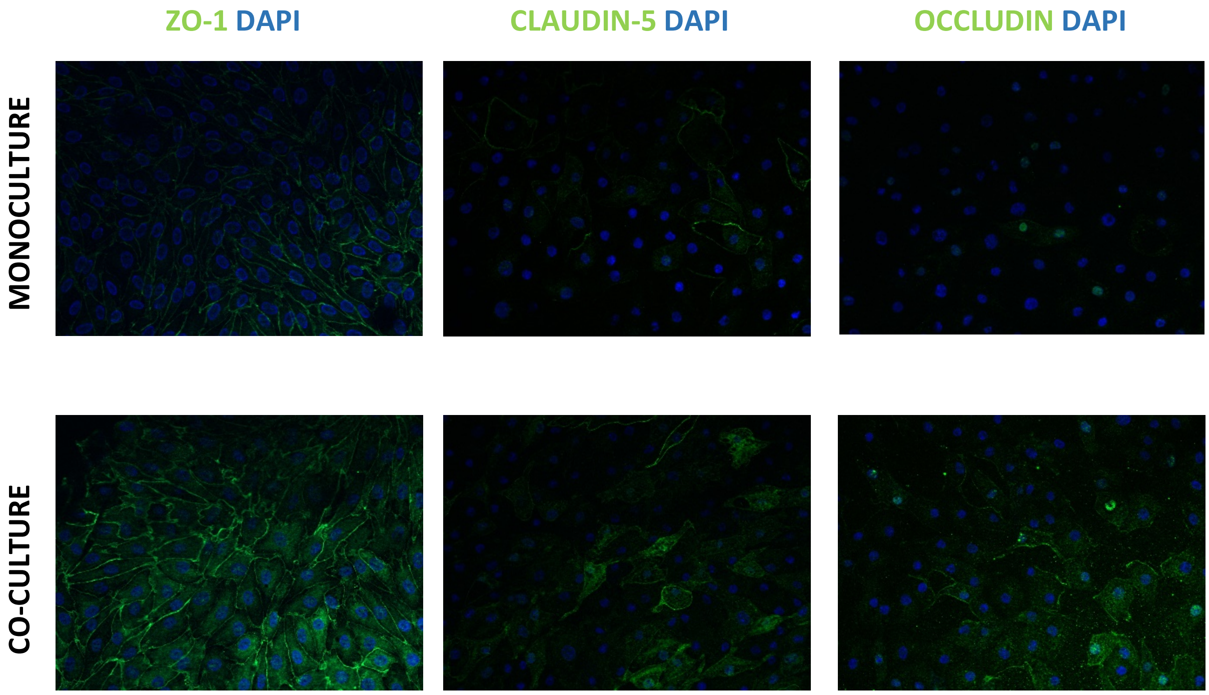

Figure S3.3: Tight junction protein expression pattern in a BBMVECs monolayer and in a BBB co-culture model. BBMVECs were fixed and stained with specific monoclonal antibodies directed respectively against ZO-1, claudin-5 and occludin. Nuclei were counterstained with DAPI.

Explanation S4: Final point criteria for animal sacrifice.

This explanation aims to describe the final point criteria for animal sacrifice. These parameters are included in the protocol number 4565 approved by Direcció General del Medi Natural, Generalitat de Catalunya and have been translated from Spanish.

**PARAMETERS OF SUPERVISION IN EXPERIMENTAL ONCOLOGY**
**Weight loss**<br>0) Normal weight<br>1) Less than 10%<br>2) Between 10 and 15% in a few days<br>3) Consistent or fast, more than 20% maintained for 72 hours.
**Physical aspect**<br>0) Normal<br>1) Dehydration, thinness. Changes in the color of the skin<br>2) Hair "not bright" "disheveled". Pallor. Cyanosis.<br>3) Encouragement. Loss of muscle mass (wasting)
**Clinical signs**<br>0) None<br>1) Hypothermia<br>2) Mucous and / or bloody secretions by any orifice. Diarrhea. Detectable hypertrophied organs (glands, spleen, liver).<br>3) Abdominal distension. Ascitic liquid volume greater than 10% by weight initial body. Breathing difficult (particularly if accompanied by nasal discharge and / or cyanosis). Caquexia.
**Alterations in behavior**<br>0) None<br>1) Incapacity to move normally<br>2) Inability to reach food / drink. Isolation of the rest of the cage animals<br>3) Unconscious or comatose. Intent to "hide" on the chip, does not respond to stimuli (dying)
**Wounds**<br>0) None<br>1) Scratches<br>2) Wounds that do not heal<br>3) Ulcerated wounds that can even be supposed. Ulcerated tumors or necrotic
**TOTAL**

When the circumstance is that there is more than one parameter with a value of 3 automatically all 3 will pass to 4.

The following monitoring criteria will be taken into account:

1. From 0 to 6 points: Individual assessment of the animal. Possible existence of pain or anguish. The use of painkillers must be considered

2. 6 to 12 points: Existence of pain or anguish. Administration of palliative analgesics.

3. From 12 to 18 points: Compulsory use of painkillers. The sacrifice of the animal must be considered.

4. From 18 to 24 points: Obligated sacrifice of the animal. The end of the procedure must be considered.

Similarly, a final point criteria will be applied at any time during the study if:

- The size of the tumor mass, although not reaching a size considered critical (3 in the assessment table), influences the other bodily functions or causes pain and / or prolonged suffering.
- Loss of body weight greater than 20% with respect to basal weight or a control.
- Ulceration or infection at the implantation site.
- Invasion of the nearby tissues by the tumor.
- Persistent self-inflammatory trauma and with an effect on the quality of life of the animal.

Explanation S5: Lentiviral plasmids preparation and culture of U87-MG cells for in vivo tumor implantation

- Lentiviral particle production

Production of viral particles was performed using human embryonic kidney cells 293T (ATCC, CRL-11268TM) grown in Dulbecco's Modified Eagle's Medium-high glucose (DMEM-hg) (Sigma, Steinheim, Germany), 10% heat-inactivated fetal bovine serum (FBS) (Sigma), 2 mM L-glutamine (Sigma), 50 units/ml penicillin/streptomycin (Sigma), and 2 mM HEPES. The day previous to transfection, $3x10^6$ cells were seeded on 10 cm^2 poly-D-lysine (Sigma) treated plates. 6 µg of the lentiviral transfer vector, pRRL-Luc-IRES-EGFP, were mixed with 2 µg of viral envelope plasmid (pMD-GVSV-G) and 4 µg of packaging construct (pCMV DR8.2) in 250 ml of 150 mM NaCl and then mixed with 48 ml of 1 mg/ml polyethylene amine (Polyscience, Warrington, PA, US) in 250 ml of 150 mM NaCl, and incubated at room temperature (RT) for 20 min. This DNA solution was then added drop wise to the plate containing the 293T cells plus medium, swirled gently and incubated for 16 h at 37ºC with 5% CO_2. The following day, the transfection solution was removed, the cells were rinsed with phosphate buffer solution (PBS) 1X and medium without FBS was added to the cells. Following a 48 h incubation the supernatant was collected, centrifuged at 2000 rpm to remove cell debris, and filtered through a 0,45 µm low protein binding filter (Corning, Bath, UK).

- Cell culture

Human glioma cells U87-MG (ATCC, HTB-14), were grown in nutrient mixture DMEM/Hams F12 HAM containing 10% heat-inactivated FBS (Sigma), 2 mM L-glutamine (Sigma) and 50units/ml penicillin/streptomycin (Sigma). U87-MG cells were infected, with pRRL-Pluc-IRES-eGFP viral stock (MOI = 20) to obtain Pluc-G-U87-MG cells, as described above. Expression of fluorescent proteins was used to select positively transduced cells.

Explanation S6: Study of the stability of polymer P.

Experimental procedure

The polymer P (batch SAG022-044) was divided in three portions and each one was kept at room temperature, 5 ºC or -21 ºC. A sample was taken at different times and analyzed by GPC to determine the molecular weight and by NMR to calculate the ratio of PEG. The ratio of PEG in the polymer was calculated from the ratio of the signal of polyester (4.06 ppm) and the signal of PEG (3.64 ppm) in the ^{1}H-NMR.

$$\% \; of \; PEG = \frac{Integral \; of \; PEG}{Integral \; of \; polyester + integral \; of \; PEG} * 100$$

The amount of acid of the polymer was determined by titration with KOH. 20 mg nanoparticles with 3wt% of the polymer 2P and 2wt% of paclitaxel were prepared. The size, pdi and zeta potential of the nanoparticles were obtained by Dynamic Light Scattering (DLS). The drug content was determined by UPLC analysis (protocol SAG-WI-035).

The stability of three different batches of polymer P was studied in order to validate the first study with one polymer. The polymer batches SAG022-050, SAG022-068 and SAG022-087 were stored at -20 ºC and samples were taken at different times. The GPC, NMR and acid content was determined as in the previous study. 20mg of nanoparticles with 3wt% of the polymer 2P and 2wt% of paclitaxel were prepared and analyzed by DLS and UPLC. The amount of PEG of the nanoparticles was calculated by the ^{1}H-NMR of the freeze-dried nanoparticles.

Results

One portion of the polymer SAG022-044 was stored at room temperature and samples were taken and analyzed at different times. The results of the experiments are summarized in Table S6.1.

Table S6.1: Characterization of the polymer P as a function of time, at RT.

Time	Mw	PDI	PEG-pol (%)	Acid (mmol/g)	Size (nm)	Pdi	Z-pot (mV)	D.C. (%)	PEG-NP (%)
3 days	16184	1.75	65	0.26	82	0.175	-25.4	1.5	56
1 week	15413	1.95	66	0.28	87	0.160	-26.1	1.7	55
3 weeks	14212	1.81	66	0.32	107	0.149	-29.4	2.1	50
1 month	13522	2.04	65	0.35	120	0.165	-33.0	2.5	48
2 months	8644	2.55	65	0.40	141	0.124	-33.9	2.0	41

The molecular weight determined by GPC decreased with time and the chromatograms are plotted in Figure S6.1. It was observed that the curve moved to lower molecular weight with time and the shape was more irregular. That indicates a degradation of the polymer. The amount of acid of the polymer increased with time (Table S5.1) because the degradation (hydrolysis) of the polymer increased the acid content. On the other hand, the amount of PEG in the polymer was constant all the time. The size and the zeta potential of the nanoparticles obtained from the polymer also increased with time. All the experiments indicated a degradation of the polymer at room temperature and for this reason the polymer P was not stable at this condition.

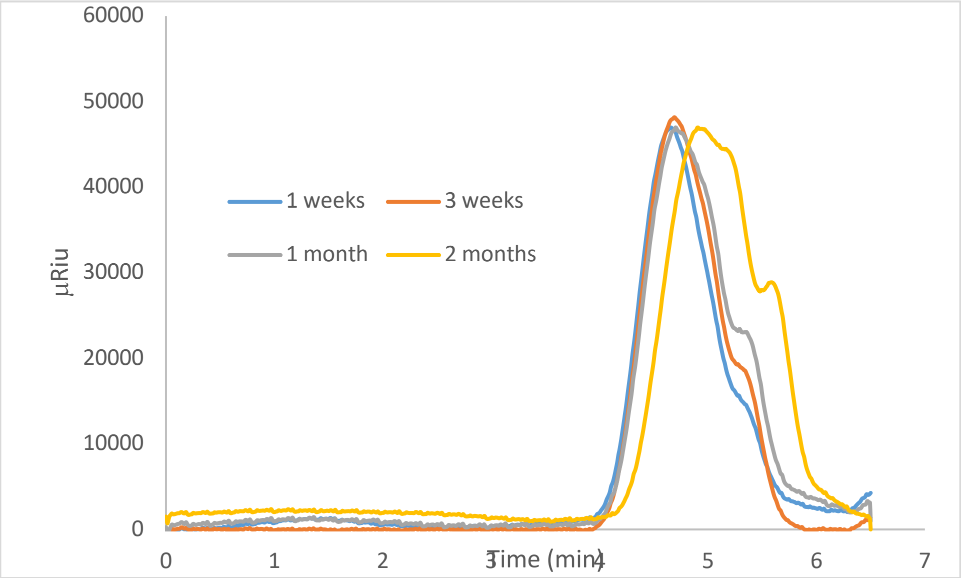

Figure S6.1: Chromatograms of the GPC of the polymer P as a function of time, at RT.

Another fraction of the polymer SAG022-044 was stored at 4 ºC and samples were taken and analyzed at different times. The results of the experiments are summarized in Table S6.2.

Table S6.2: Characterization of the polymer P as a function of time, at 4ºC.

Time	Mw	PDI	PEG-pol (%)	Acid (mmol/g)	Size (nm)	Pdi	Z-pot (mV)	D.C. (%)	PEG-NP (%)
3 days	16537	1.80	65	0.26	78	0.166	-23.4	1.7	56
1 week	16141	1.98	65	0.26	82	0.172	-24.6	2.0	56
3 weeks	16469	1.73	65	0.26	83	0.181	-27.7	1.9	56
1 month	16146	1.90	65	0.26	81	0.180	-27.0	1.7	55
2 months	15616	1.84	65	0.28	83	0.164	-26.2	1.8	55
3 months	15566	1.84	65	0.28	84	0.160	-21.7	2.2	54
4 months	15382	1.79	65	0.30	91	0.189	-30.1	1.4	54

The molecular weight decreased after 3 months but the chromatograms were quite similar for all the samples (Figure S6.2). The amount of acids increased slightly after 2 months but less than the sample stored at room temperature. The nanoparticles obtained from the polymer showed similar size and zeta potential up to 3 months. In this case, the experiments indicated that polymer P was reasonably stable at 4 ºC for a short time storage.

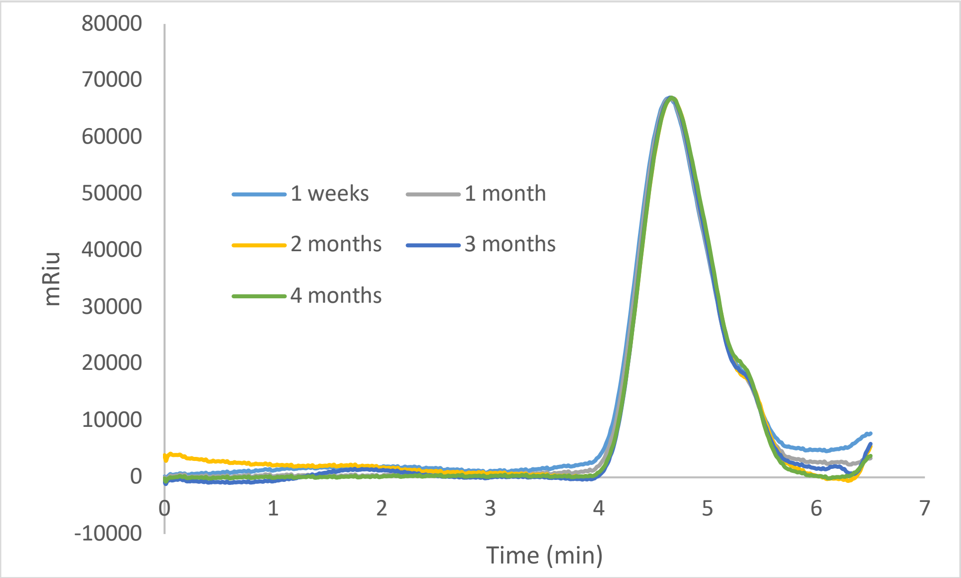

Figure S6.2: Chromatograms of the GPC of the polymer P as a function of time, at 4ºC.

The third fraction of the polymer SAG022-044 was stored at -20 ºC and samples were taken and analyzed at different times. The results of the experiments are summarized in Table S6.3.

Table S6.3: Characterization of the polymer P as a function of time, at -20ºC.

**Time**	**Mw**	**PDI**	**PEG-pol** (%)	**Acid** (mmol/g)	**Size** (nm)	**Pdi**	**Z-pot** (mV)	**D.C.** (%)	**PEG-NP** (%)
3 days	16608	2.03	65	0.26	80	0.191	-21.8	1.9	56
1 week	15957	1.73	66	0.26	81	0.172	-24.0	1.9	56
3 weeks	15907	1.63	65	0.26	79	0.175	-25.6	2.1	55
1 month	16235	1.89	65	0.26	80	0.148	-27.0	2.2	56
2 months	16038	1.74	65	0.26	80	0.151	-25.4	2.2	56
3 months	16501	1.72	65	0.26	77	0.146	-22.0	2.3	56
4 months	16310	1.75	65	0.26	78	0.176	-27.3	1.3	56

The molecular weight for the polymer stored at -20 ºC was quite stable in time and the chromatograms were very similar (Figure S6.3). The acid amount was constant all the time and the nanoparticles had quite similar size and zeta potential. This data indicated that polymer P was enough stable at -20 ºC in the period of the study.

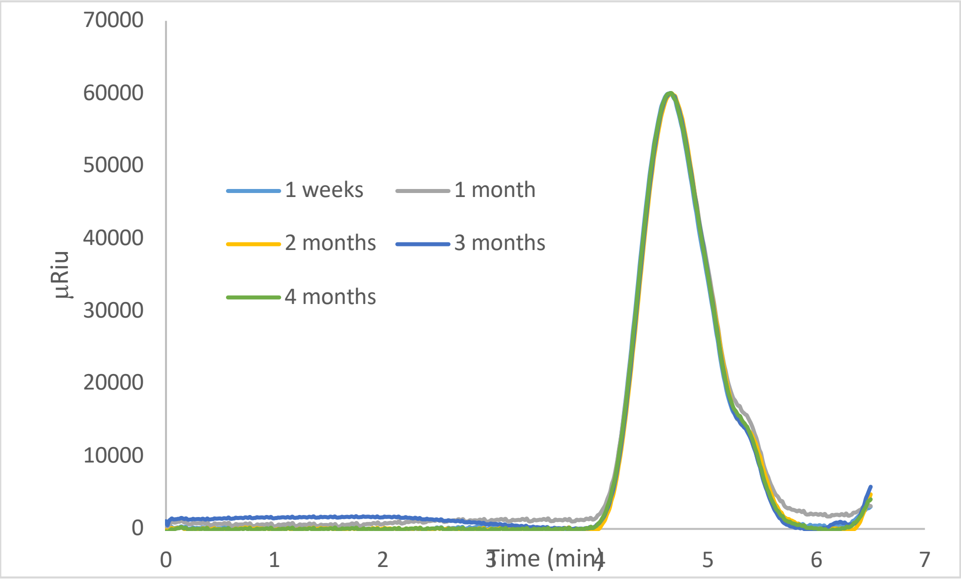

Figure S6.3: Chromatograms of the GPC of the polymer P as a function of time, at -20ºC.

To complete the study, three different batches of polymer P (SAG022-050, SAG022-068 and SAG022-87) were kept at -20 ºC and samples were analyzed at different times. The results of the experiments are summarized in Table S6.4.

Table S6.4: Characterization of three batches of the polymer P as a function of time, at -20ºC.

Polymer	Time	Mw	PDI	PEG-pol (%)	Acid (mmol/g)	Size (nm)	Pdi	Z-pot (mV)	PEG-NP (%)	D.C. (%)
SAG022-050	Initial	13400	1.7	55	0.35	119	0.119	-31.9	45	1.7
	2 months	-	-	54	0.41	127	0.127	-31.1	45	2.0
	3 months	13300	1.6	55	0.42	126	0.166	-31.8	45	2.1
	4 months	13200	1.6	55	0.40	129	0.173	-33.6	44	1.8
SAG022-068	Initial	-	-	59	0.37	126	0.142	-33,0	46	1.6
	1 month	13500	1.7	59	0.38	127	0.129	-34.4	47	1.8
	2 months	13700	1.6	59	0.38	128	0.132	-39.7	46	2.3
	3 months	13600	1.8	59	0.38	124	0.158	-32.8	46	2.2
SAG022-087	Initial	-	-	62	0.33	106	0.157	-35.1	49	1.9
	2 weeks	-	-	62	0.34	106	0.163	-33.3	49	2.1
	1 months	14700	1.7	62	0.35	109	0.167	-33.7	49	3.1
	2 month	14900	1.7	63	0.34	105	0.166	-30.5	50	2.2

The molecular weight and the amount of acids were quite similar for each polymer in the period of study. The chromatograms of the polymer at the end time of the study were very similar and degradation was not detected (Figure S6.4). On the other hand, the nanoparticles obtained from the polymer had not change in size or zeta potential along the time.

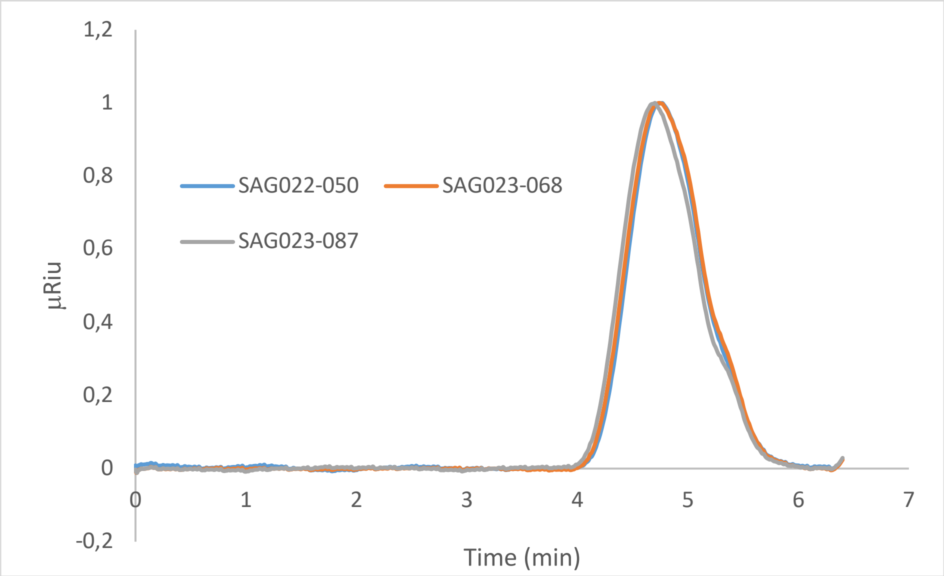

Figure S6.4: Chromatograms of the GPC of three batches of the polymer P at -20ºC, at the end of the study.

Conclusions

The polymer P was kept at -20 ºC and no changes were observed in the capacity to obtain nanoparticles from it at least for 4 months. This is the most appropriate temperature for a long storage of the polymer.

The polymer could be stored at 4 ºC for 2 months. It is important to avoid the storage of the polymer at room temperature, because at this temperature the degradation of the polymer is faster.

Explanation S7: Study of the stability of polymer 2P.

Results

Three batches of polymer 2P SAG023-033, SAG023-070 and SAG023-084 were stored at -20 ºC and samples were taken and analyzed by NMR at different times. The results of the experiments are summarized in Table S7.1.

Table S7.1: Characterization of three batches of the polymer 2P as a function of time.

Polymer	Time	Polyester (% of peaks)	Peptide (%)
SAG023-070	Initial	2.3	60
	2 weeks	2.2	61
	3 weeks	2.0	61
	1 month	2.2	58
	6 weeks	2.1	55
SAG023-084	Initial	2.0	61
	1 week	1.8	61
	2 week	2.0	62
	5 weeks	2.0	59
SAG023-091	Initial	1.9	55
	1 month	2.0	58
	5 weeks	1.9	62

A small reduction in the amount of peptide for the polymer SAG023-070 after 1 month was observed. On the other hand, the ratio of polyester had not a considerable variation. This data could indicate a small degradation of the polymer SAG023-070 after 1 month. But the other polymers SAG023-084 and SAG023-091 seem to be more stable.

The polymer 2P was dissolved in dimethyl sulfoxide (DMSO) for the synthesis of the nanoparticles. For this reason, the stability of the polymer 2P was also study in solution of DMSO. A solution of polymer 2P of 12 mg/mL in DMSO was stored at -20 ºC and samples were taken and analyzed by NMR at different times. The results of the experiments are summarized in Table S7.2.

Table S7.2: Characterization of the polymer 2P dissolved in DMSO as a function of time.

Polymer	Time	P* (% of peaks)	Peptide (%)
	Initial	1.9	55
	1 week	2.0	52
**SAG023-091**	3 weeks	2.1	51
	1 month	2.1	56
	6 weeks	1.9	55

The ratio of polyester and PEG in the polymer had no variation with time and the percentage of peptide was quite similar in the period of study. We can say that the solution of polymer 2P in DMSO was stable at – 20 ºC after 6 weeks at the concentration of study.

Conclusions

The polymer 2P can be stored in solid state or solution in dimethyl sulfoxide (12 mg/mL) at -20 ºC at least for 6 weeks.

Explanation S8: PTX release studies

Previous work on paclitaxel (PTX) release profile characterization suggested that the widely used dialysis membrane method was not appropriate due to poor solubility of PTX in aqueous media, and crossing problems through the dialysis membrane when a hydrotropic agent is used for its enhancement. Thus, an alternative method was applied based on nanoparticles filtration and centrifugation. Experiments were performed with the presence of human serum albumin (HSA) and without it, in order to evaluate differences between drug release profiles without and with the presence of proteins.

Briefly, 10 mL of nanoparticles (6 mg/mL, drug content: 1.45%) were placed in a Falcon tube in PBS media or HSA (6 mg/mL in PBS) solution. Both nanoparticles suspension were incubated at 37°during 1 hour, 4 hours and 24 hours, and samples were removed at the corresponding time points. All samples were analyzed by UPLC (Waters Acquity UPLC H-Class), by using the method described in Explanation S2.

Recovery of PTX was between 70% and 90%. The released fraction of the drug is represented in Figure S7.1. Drug release profile shows a normal increasing tendency with time. While drug release performed in PBS resulted in more than 25% drug release at 24 hours, this value is decreased to less than 15% when test is performed in HSA, suggesting than HSA takes an active role in drug release event.

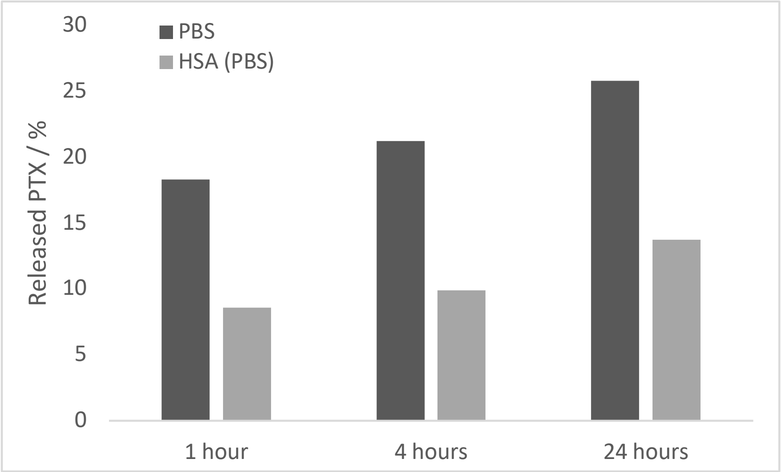

Figure S8.1:. PTX release from nanoparticles at 1 hour, 4 hours and 24 hours, with and without HSA.

Explanation S9: Nanoparticles long-term stability study

Schematic working conditions of the nanoparticles freezing

In Figure S9.1, the conditions at which nanoparticles have been submitted to find out the best storing condition are schematized.

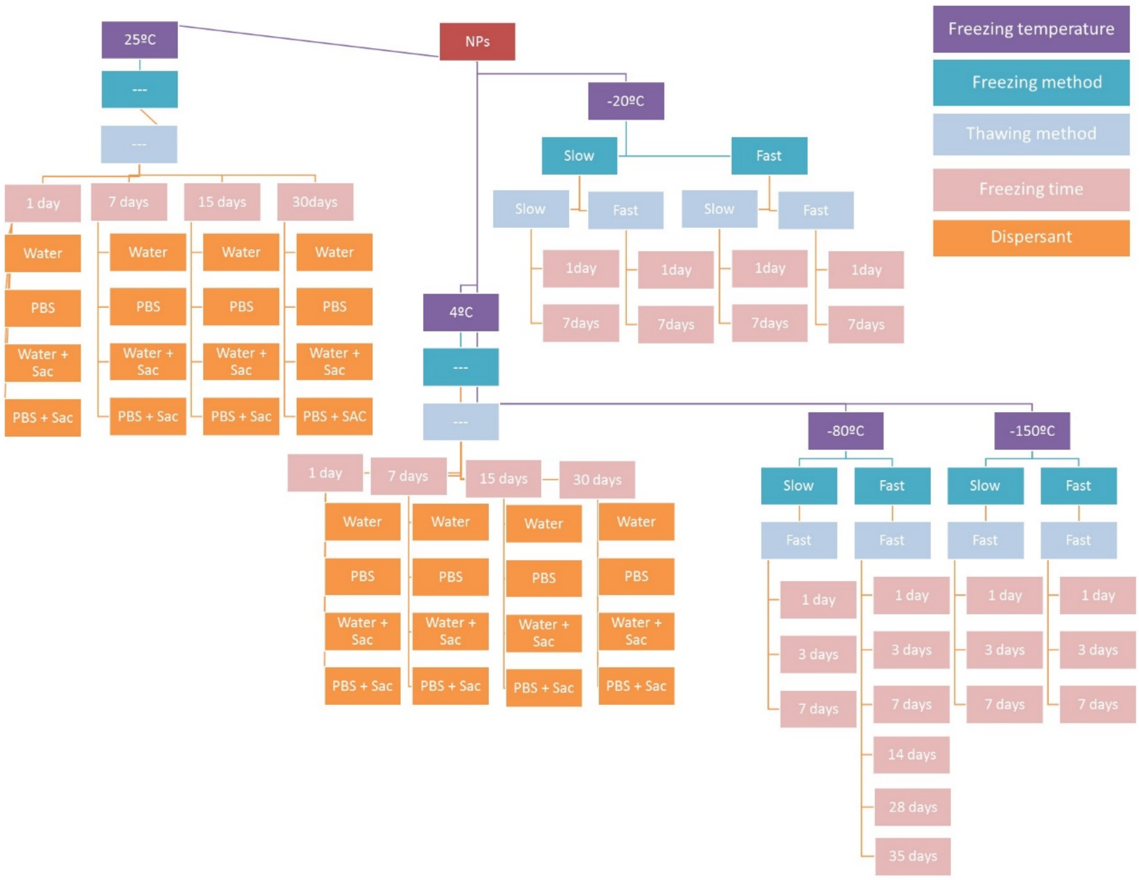

Figure S9.1: Schematic representation of the conditions at which nanoparticles have been submitted to find out the best storing condition.

Results

1) *NP stability to freeze-drying*

Many studies have described freeze-drying as an appropriate methodology to store nanoparticles in solid state. For this reason, in the present work, nanoparticles were also

freeze-dried after a freezing step at -20ºC and -80ºC, and then, their sizes were measured. Results are presented in Table S9.1.

Table S9.1: Hydrodynamic diameter (in nm) of nanoparticle dispersions after being freeze-dried (freezing step at -20 or -80ºC for 1, 4 and 24hours and thawing at room temperature)

			Freezing time		
		**Sonication time**	**1h**	**4h**	**24h**
**Freezing temperature**	**-20ºC**	**0 min**	> 500 nm	> 500 nm	> 1000 nm
		**5 min**	> 300 nm	> 150 nm	> 250 nm
		**10 min**	> 300 nm	> 170 nm	> 400 nm
	**-80ºC**	**0 min**	> 1000 nm	> 1000 nm	> 800 nm
		**5 min**	> 700 nm	> 300 nm	> 800 nm
		**10 min**	> 700 nm	> 600 nm	> 1000 nm

As Table S9.1 shows, independently of the freezing time and freezing temperature, nanoparticles were not stable at after freeze-drying. Their sizes increased dramatically, which was expected according to their visual appearance (they showed a more turbid and viscous aspect). Sonication for up to 10 minutes after thawing did not improve the results obtained, indicating that these nanoparticles could not undergo a freeze-drying process.

2) *NP stability as a function of the temperature*

a. *At 25ºC and 4ºC as a function of dispersant media*

Nanoparticles were dispersed either in water or PBS, without and with 10wt% of sucrose. Then, they were stored at 25ºC and 4ºC. At specific time points, their size, PDI and surface charge were measured. In addition, the pH of the media was also followed as a function of time, since it could be an indication of the nanoparticles hydrolysis. Results are presented in the following Figures, separately to facilitate the discussion.

First, the results of the pH as a function of time are described in Figure S9.2. As expected, the pH of the nanoparticle dispersions depended on their dispersant: when dispersed in water (Figure S8.2a), nanoparticles suffered an initial pH decrease down to pH around 4 – 4.5. However, after around 35 days, the pH suffered another decrease, independently on the storing temperature and on the presence of sucrose. Nevertheless, nanoparticles dispersed in 10wt% sucrose, at 4ºC, showed the highest stability. In contrast, when nanoparticles were dispersed in PBS (Figure S8.2b), due to the buffer nature of it, the pH of the dispersion was maintained constant at pH around 6.5 – 7, for the whole period of time studied.

It is worth noting that further pH decreases, specifically when nanoparticles are dispersed in water are not expected, because nanoparticles are composed of carboxylic acids, which maximum pH decreases down to around 4.

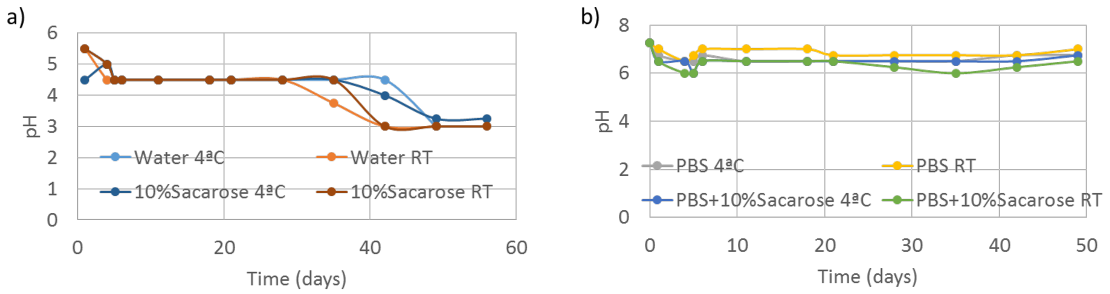

Figure S9.2: pH of nanoparticle dispersions as a function of time, suspended in a) water or b) PBS, without and with 10wt% sucrose, at 25ºC or 4ºC.

As Figure S9.3 shows, initially, nanoparticles showed hydrodynamic diameters around 125 nm. All NPs formulations maintained their sizes over time independently on the dispersion media and the storing temperature up to 20 days. Then, a size increase was observed for all nanoparticles, but in a higher extent when they were stored at RT. Therefore, nanoparticles are stable in terms of size for at least, 20 days and the most stable nanoparticles are those preserved at 4ºC, dispersed in water + 10wt% sucrose (up to 30 days).

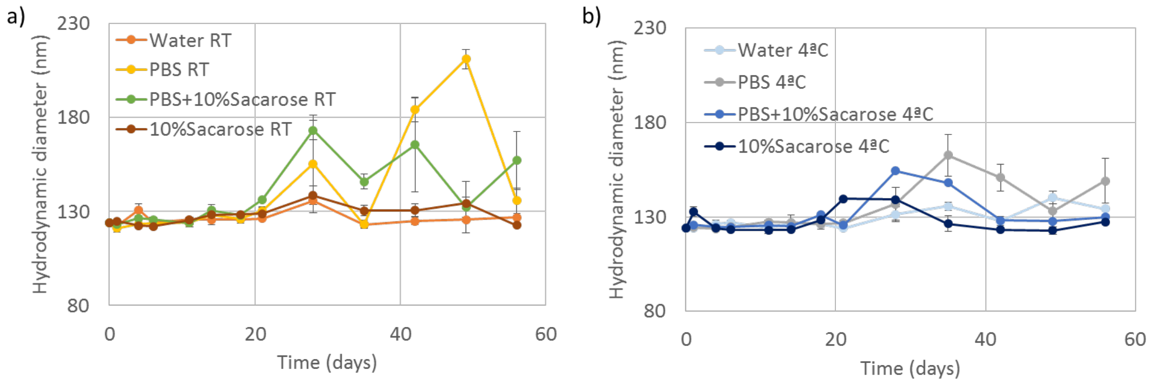

Figure S9.3: Hydrodynamic diameter (nm) of nanoparticle dispersions as a function of temperature, at a) room temperature (RT) or b) 4ºC, without and with 10wt% sucrose, dispersed in water or PBS.

Related with the size measurements, the PDI of nanoparticles was also followed as a function of time (Figure S9.4), resulting in PDI values that confirmed size distributions as monodisperse, at least, during the first 20 days, independently on the storage conditions. Then, nanoparticles dispersed in water suffered a slight polydispersity increase, while those dispersed in PBS suffered a higher increase on the polydispersity (Figure S8.4).

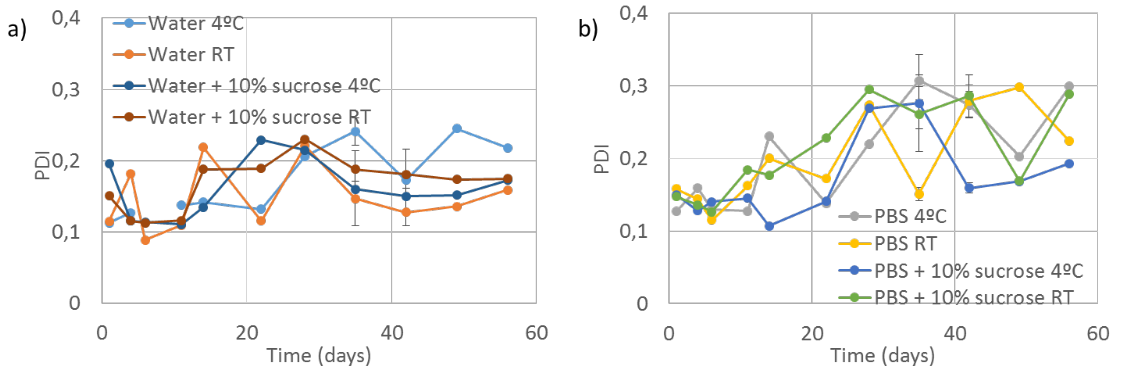

Figure S9.4: PDI of nanoparticle dispersions as a function of dispersant, dispersed in a) water or b) PBS, without and with 10wt% sucrose, at 4ºC or RT.

Regarding surface charge, as Figure S9.5 shows, after some initial variations, it is more or less constant over time, but it depended on the dispersant media more than on the temperature. When nanoparticles were dispersed in water, they showed surface charges around -5 mV, while the surface charge was around -11 mV when they were dispersed in PBS. The increase in absolute values of the surface charge when nanoparticles are suspended in PBS could be due to their interaction with the electrolytes contained in this buffer, which can also be included surrounding nanoparticles and contributing to their surface charge. At longer time points, nanoparticles dispersed in PBS at RT, independently on the presence of glucose, tended to destabilize (Figure S9.5b).

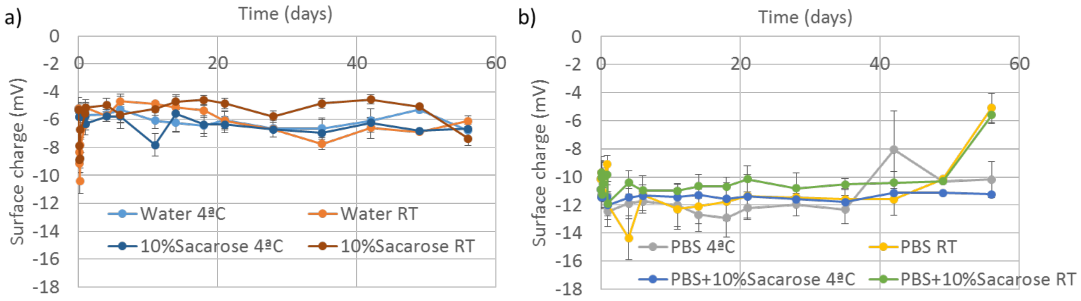

Figure S9.5: Surface charge of nanoparticle dispersions as a function of dispersant, dispersed in a) water or b) PBS, without and with 10wt% sucrose, at 4ºC or RT.

To sum up this section, it can concluded that nanoparticles dispersed in water + 10wt% sucrose have the highest stability. Therefore, this dispersant medium will be selected for further storage experiments. At 4ºC, as expected, nanoparticles were more stable than at room temperature.

b. *Stability of frozen NP as a function of freezing/thawing speed and freezing temperature*

Freezing / thawing speed is a parameter defined in previous bibliography to influence the stability of nanoparticles once frozen. For this reason, diverse conditions of freezing / thawing were tested. First, the studies were intended to achieve nanoparticles freezing and **storage at -20ºC**. Nanoparticles were frozen in a fast way (directly frozen to -20ºC) and in a slow way (decreasing the temperature 1ºC/min until -20ºC). Then, they were thawn in a fast way (directly at room temperature, let thawing for 30 min) and in a slow way (30 min at 4ºC followed by 30 more min at room temperature).

After this procedure, size, PDI and surface charge were characterized after 1 day and 7 days of freezing. In addition, the paclitaxel concentration was also quantified. Results are presented in Table S9.2.

Table S9.2: Hydrodynamic diameter (in nm), including PDI; surface charge (in mV) and concentration of paclitaxel (in mg/mL) of nanoparticles after freezing at -20ºC and thawing at room temperature using different methodologies, for 1 and 7 days. Values in red indicate results markedly deviated from those specified in the specifications.

			Thawing method							
			Fast				Slow			
		Storage time	Size (nm)	PDI	Zpot (mV)	[PTX] (mg/mL)	Size (nm)	PDI	Zpot (mV)	[PTX] (mg/mL)
Freezing method	Slow	1 day	132 nm	0,134	0,3 mV	0,976	133 nm	0,13	-3,54mV	1,398
		7 days	249 nm	0,402	-3,1 mV	0,965	220 nm	0,301	-2,85 mV	0,967
	Fast	1 day	130 nm	0,133	-3,3 mV	1,482	129 nm	0,136	-3,72mV	1,580
		7 days	214 nm	0,268	-3,1 mV	1,062	186 nm	0,219	-3,29 mV	1,144

The initial size of these nanoparticles was around 111 nm, with a PDI around 0.115, their surface charge around -4 mV and the paclitaxel concentration around 1 mg/mL. After 1 day of freezing, independently on thawing and freezing method, nanoparticles sizes slightly increased up to around 130 nm. The PDI was maintained in the range to define nanoparticles as monodisperse; the surface charge, except for one value, slightly decreased in absolute value to around -3mV and the concentration of paclitaxel was maintained as the initial one. Therefore, these results seem to indicate that, using the

conditions defined in this experiment, nanoparticles were stable, for at least 1 day, at -20ºC.

However, after 7 days frozen, results were very different, independently of the freezing / thawing method used. Nanoparticles sizes increased notably up to diameters around 200 nm, thus producing an increase on the PDI value. Although the surface charge and paclitaxel concentration seemed to be maintained, these nanoparticles were not stable to freezing 7 days.

Since at -20ºC nanoparticles were not stable for long periods of time, their **stability at -80ºC and -150ºC** was also tested, firstly during one week. The same parameters were tested. Initially, these nanoparticles showed hydrodynamic diameters of around 120nm, PDI typically lower than 0.2, surface charges between -2 and -6 mV and concentrations of paclitaxel around 1mg/mL.

As Table S9.3 shows, nanoparticles sizes, PDI, surface charges and paclitaxel concentration were maintained similar to those found initially, independently on the freezing temperature and the freezing method, for at least, one week.

Table S9.3: Hydrodynamic diameter (in nm), including PDI; surface charge (in mV) and concentration of paclitaxel (in mg/mL) after freezing at -80 and -150ºC and thawing at room temperature for 1, 3 and 7 days. Values in red indicate results markedly deviated from those specified in the specifications.

			Freezing temperature							
			-80ºC				-150ºC			
		Storage time	Size (nm)	PDI	Zpot (mV)	[PTX] (mg/mL)	Size (nm)	PDI	Zpot (mV)	[PTX] (mg/mL)
Freezing method	Fast	1 day	120 nm	0,169	-4,4 mV	1,07	116 nm	0,132	-2,9 mV	0,88
		3 days	120 nm	0,154	-3,9 mV	1,06	116 nm	0,129	-3,8 mV	0,97
		7 days	125 nm	0,212	-4,6 mV	1,05	117 nm	0,152	-2,9 mV	0,98
	Slow	1 day	121 nm	0,142	-5,2 mV	0,98	121 nm	0,136	-2,7 mV	0,82
		3 days	122 nm	0,154	-4,3 mV	1,07	121 nm	0,150	-3,4 mV	1,11
		7 days	124 nm	0,165	-4,5 mV	1,11	121 nm	0,136	-3,9 mV	0,97

The results using a fast freezing are very similar between -80ºC and -150ºC, therefore, -80ºC was chosen to perform further studies on nanoparticles freezing. Accordingly, nanoparticles physicochemical properties after **freezing at -80ºC**, using a **fast freezing** were determined for a **longer storage time**. As Table S8.4 shows, all the studied

parameters (size, PDI, z potential and PTZ concentration) kept constant as a function of time for, at least, 50 days (Table S9.4). Therefore, these nanoparticles can be stored frozen at -80ºC for nearly 2 months.

Table S9.4: Hydrodynamic diameter (in nm), including PDI; surface charge (in mV) and concentration of paclitaxel (in mg/mL) in nanoparticle dispersions after freezing at -80ºC and thawing at room temperature, as a function of time.

Time (days)	Size (DLS)	Pdi	Z-Pot (mV)	[PTX] (mg/mL)
0	120 nm	0.169	- 4.53	1.07
3	120 nm	0.154	- 3.49	1.06
7	116 nm	0.215	- 3.51	1.06
14	112 nm	0.154	- 3.26	1.15
21	114 nm	0.196	- 3.27	1.09
28	120 nm	0.214	- 3.76	1.02
35	120 nm	0.215	- 3.28	1.09
42	121 nm	0.211	- 3.48	0.99
50	126 nm	0.263	- 4.56	1.09

In addition, since -80ºC seemed an optimal temperature to freeze nanoparticles, but -20ºC did not, it was tried to perform a **first freezing at -80ºC** for 1h, 4h and 24h, **followed by the nanoparticles freezing at -20ºC**. Nanoparticles characterization was performed at various time-points, as specified in Table S9.5. As this Table shows, all parameters kept constant for, at least, 11 days (longer times under study). Therefore, after a short freezing at -80ºC, nanoparticles can be frozen at -80ºC.

Table S9.5: Hydrodynamic diameter (in nm), including PDI; surface charge (in mV) and concentration of paclitaxel (in mg/mL) in nanoparticle dispersions after freezing at -80 and -20ºC and thawing at room temperature, as a function of time.

Time at -80ºC (hours)	Time at -20ºC (days)	Size (DLS)	Pdi	Z-Pot (mV)	[PTX] (mg/mL)
**1**	1	118 nm	0.138	- 4.54	0.59
	4	126 nm	0.182	- 4.21	0.59
	6	119 nm	0.115	- 4.65	0.59
	11	121 nm	0.124	- 5.69	0.58
**4**	1	120 nm	0.139	- 4.10	0.62
	4	120 nm	0.121	- 4.85	0.59
	6	123 nm	0.098	- 4.15	0.61
	11	124 nm	0.143	- 5.70	0.62
**24**	1	119 nm	0.199	- 4.04	0.63
	4	121 nm	0.134	- 3.38	0.58
	6	121 nm	0.126	- 4.35	0.61
	11	125 nm	0.130	- 5.00	0.59

3) *Stability to filtration*

Table S9.6 shows the results of an example of the filtration of a nanoparticles big batch through 0.22 μm and 0.45 μm and its post characterization. As this table shows, nanoparticle size, PDI and zeta potential was maintained constant after nanoparticle filtration. Therefore, the filtration did not produced changes in nanoparticles.

Table S9.6: Schematic representation of the conditions at which nanoparticles have been submitted to study the filtration.

Batch of NPs	Filtration (pore size)	Hydrodynamic diameter (nm)	PDI	Z-Pot (mV)	[PTX] (mg/mL)
SAG022-052<br>P: SAG022-050<br>2P: SAG020-156	Unfiltered	118 nm	0.142	-4.8 mV	1.2 mg/mL
	0.22 μm	118 nm	0.142	-4.2 mV	1.2 mg/mL
	0.45 μm	118 nm	0.147	-4.0 mV	1.2 mg/mL

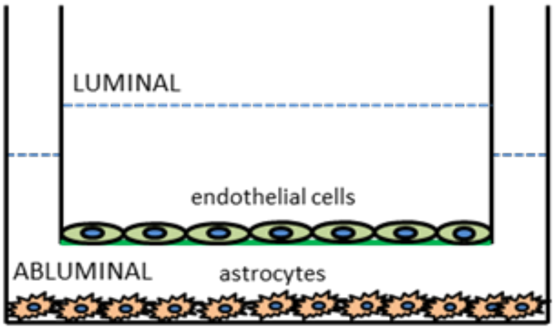

Figure S1: Schematic representation of the in vitro BBB models. BBMVECs are cultured on the collagen coated insert surface and astrocytes are grown below, on the bottom of the multi-well plate.

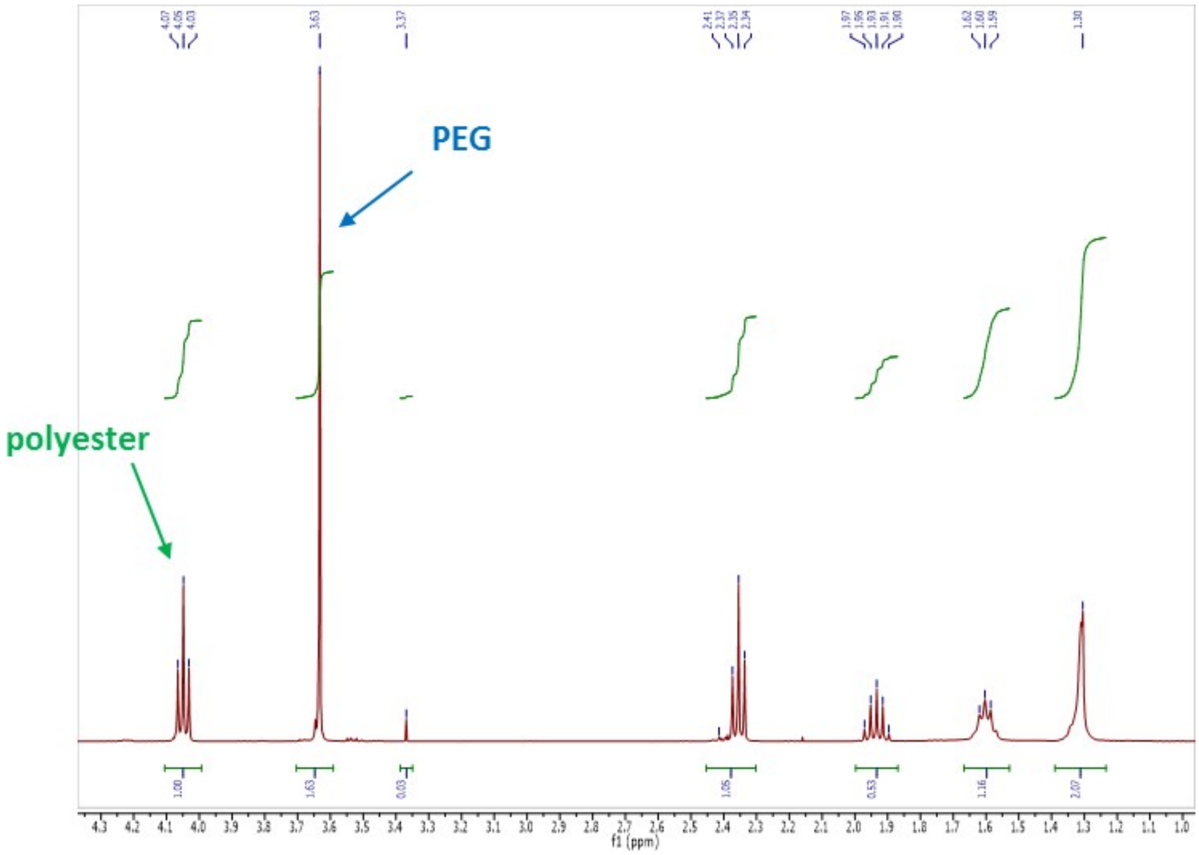

Figure S2: ^{1}NMR of an example batch of the P co-polymer.

As Figure S2 shows, a large peak appears at 3.6 ppm, which is attributed to PEG (methylene groups); as well as a multiplet at 4.1 ppm, attributed to the CH2 groups of 1,8-octanediol. The ratio of these two peaks confirmed a PEG content of around 60% for all batches studies (Table 1 from the main paper).

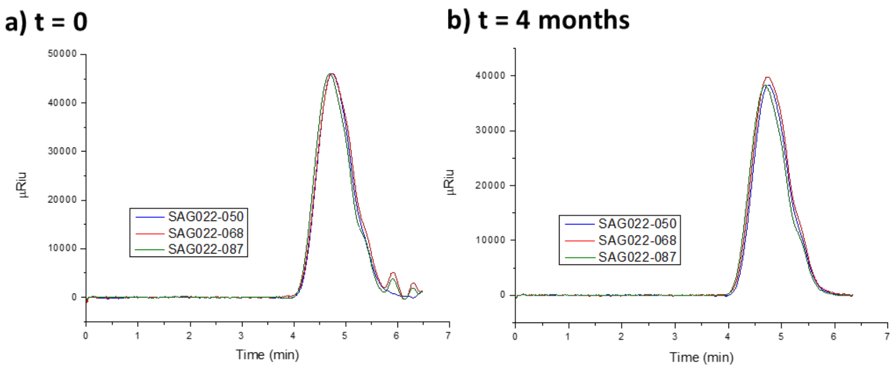

Figure S3: GPC of three big batches of the polymer P, at a) initial preparation time (t=0) and b) after 4 months stored at -20ºC.

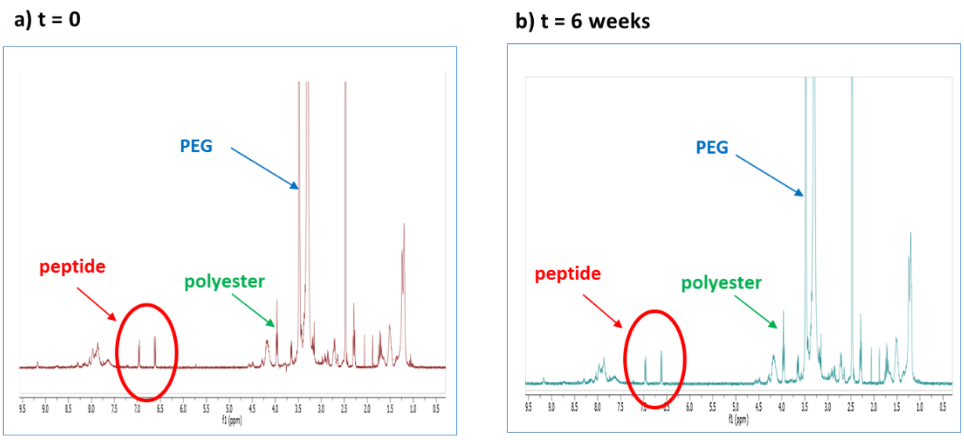

Figure S4: ^{1}NMR of an example batch of the polymer 2P, at a) initial preparation time (t=0) and b) after 6 weeks stored at -20ºC.

Apart from the PEG and polyester peaks, a double peak around 6.5 – 7 ppm was attributed to the peptide.

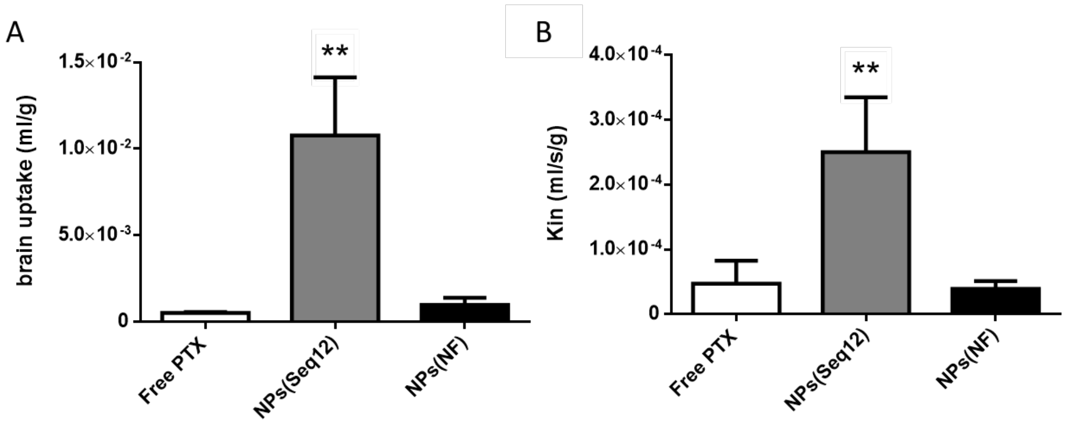

Figure S5: A - Brain uptake and B - K_{in} parameter of free PTX and PTX-NPs, funcionalized with Seq12 peptide NPs(Seq12) and without any functionalization NPs(NF). **p<0.01.

With this Figure, it is demonstrated that it is required the Seq12 peptide to achieve the crossing of the BBB and PTX entry and uptake to the brain. Therefore, it is demonstrated that the brain uptake is due to the presence of Seq12 peptide and not only the effect of PTX encapsulation in NPs.

Table S1: Physicochemical characterization of three independent batches of polymer P.

Parameter	**Batch 1:** SAG022-050	**Batch 2:** SAG022-068	**Batch 3:** SAG022-087
**Molecular weight** (g/mol) *by GPC*	13400	13500	14700
**Acid content** (mmol/g)	0.35	0.37	0.33
**PEG content** (%) *by H-RMN*	55	59	62
**Resulting NP sizes** (nm) *by DLS*	119	126	106

Table S2: Physicochemical characterization of three independent batches of polymer 2P.

Parameter	**Batch 1:** SAG023-070	**Batch 2:** SAG023-084	**Batch 3:** SAG023-091
**Polyester peaks** (%) *by RMN*	2.3	2.0	1.9
**Peptide peaks** (%) *by RMN*	60	61	55

Table S3: Physicochemical characterization of three independent small batches of nanoparticles.

Parameter	**Batch 1:** SAG027-022	**Batch 2:** SAG027-035	**Batch 3:** SAG027-036
**Hydrodynamic diameter** (nm) *by DLS*	120	120	126
**PDI**	0.119	0.116	0.116
**Surface charge** (mV) *by electrophoretic mobility*	-5.2	-4.6	-4.3
**PEG content** (%) *by H-RMN*	44	45	43
**D.C.** (%)	2.2	2.1	2.2
**PTX concentration** (mg/mL)	0.27	0.26	0.28

Table S4: Physicochemical characterization of three independent big batches of nanoparticles.

Parameter	**Batch 1:** SAG022-078	**Batch 2:** SAG027-052	**Batch 3:** SAG027-060
**Hydrodynamic diameter** (nm) *by DLS*	106	108	100
**PDI**	0.113	0.094	0.117
**Surface charge** (mV) *by electrophoretic mobility*	-4.8	-4.2	-4.0
**PEG content** (%) *by H-RMN*	44	42	46
**D.C.** (%)	1.5	1.6	1.5
**PTX concentration** (mg/mL)	1.04	1.12	1.05

Table S5: Nanoparticle induced hemolysis (%). n=6replicates/sample. Note that PTX concentrations are much higher than in in vitro experiments because in hemolysis experiments, the expected injected concentration must be tested, and it is always higher in vivo than in vitro.

Sample	Hemolysis percentage
Negative control (PBS)	0
Positive control	100
NP at 60 mg/mL (PTX=11.24μM)	15.38
NP at 6 mg/mL (PTX=1.124μM)	1.42
